# The evolution of heat shock protein sequences, cis-regulatory elements, and expression profiles in the eusocial Hymenoptera

**DOI:** 10.1186/s12862-015-0573-0

**Published:** 2016-01-19

**Authors:** Andrew D. Nguyen, Nicholas J. Gotelli, Sara Helms Cahan

**Affiliations:** Department of Biology, University of Vermont, Burlington, VT 05405 USA

**Keywords:** Heat shock proteins, Heat shock response, Heat shock elements, Thermal tolerance, Gene expression, Cis-regulation, Comparative genomics

## Abstract

**Background:**

The eusocial Hymenoptera have radiated across a wide range of thermal environments, exposing them to significant physiological stressors. We reconstructed the evolutionary history of three families of Heat Shock Proteins (Hsp90, Hsp70, Hsp40), the primary molecular chaperones protecting against thermal damage, across 12 Hymenopteran species and four other insect orders. We also predicted and tested for thermal inducibility of eight Hsps from the presence of cis-regulatory heat shock elements (HSEs). We tested whether Hsp induction patterns in ants were associated with different thermal environments.

**Results:**

We found evidence for duplications, losses, and cis-regulatory changes in two of the three gene families. One member of the Hsp90 gene family, *hsp83*, duplicated basally in the Hymenoptera, with shifts in HSE motifs in the novel copy. Both copies were retained in bees, but ants retained only the novel HSE copy. For Hsp70, Hymenoptera lack the primary heat-inducible orthologue from *Drosophila melanogaster* and instead induce the cognate form, *hsc70-4*, which also underwent an early duplication. Episodic diversifying selection was detected along the branch predating the duplication of *hsc70-4* and continued along one of the paralogue branches after duplication. Four out of eight Hsp genes were heat-inducible and matched the predictions based on presence of conserved HSEs. For the inducible homologues, the more thermally tolerant species, *Pogonomyrmex barbatus*, had greater Hsp basal expression and induction in response to heat stress than did the less thermally tolerant species, *Aphaenogaster picea*. Furthermore, there was no trade-off between basal expression and induction.

**Conclusions:**

Our results highlight the unique evolutionary history of Hsps in eusocial Hymenoptera, which has been shaped by gains, losses, and changes in cis-regulation. Ants, and most likely other Hymenoptera, utilize lineage-specific heat inducible Hsps, whose expression patterns are associated with adaptive variation in thermal tolerance between two ant species. Collectively, our analyses suggest that Hsp sequence and expression patterns may reflect the forces of selection acting on thermal tolerance in ants and other social Hymenoptera.

**Electronic supplementary material:**

The online version of this article (doi:10.1186/s12862-015-0573-0) contains supplementary material, which is available to authorized users.

## Background

Heat stress causes proteins to lose stability, misfold, and form aggregates, which can impair function and reduce organismal fitness [1–4]. To cope with macromolecular damage, the heat shock response (HSR) transcriptionally up-regulates thermally responsive genes such as heat shock proteins (Hsps), which maintain proteostasis by refolding or degrading denatured proteins and preventing aggregations [1, 2, 5]. Hsps are a set of highly conserved molecular chaperone proteins of diverse multigene families, named after their molecular weight (Hsp90, Hsp70, Hsp60, Hsp40, and small Hsps) [6, 7].

Although Hsps as a group are highly conserved, diversity within each Hsp gene family reflects evolutionary gains and losses of gene copies [8, 9]. Each Hsp protein family includes paralogues localized to different subcellular compartments (cytosol, endoplasmic reticulum, or mitochondria) that participate in housekeeping functions and/or respond to environmental stress [10–12]. For heat-inducible forms, the transcribed heat shock factors (HSF), bind to cis-regulatory elements known as heat shock elements (HSEs) and up-regulate Hsp transcription [13–15]. Patterns of variation in Hsp gene expression among taxa include expansion of additional Hsp genes [16] and shifts in the arrangement and position of HSE elements [14, 17, 18]. Among taxa, both the level of constitutive expression and the magnitude of Hsp induction are associated with adaptive variation in upper thermal limits [19–22]. Gene structure may also play a role in Hsp expression, but has not been well-studied. For example, genes with introns allow for more mRNA accumulation than do intronless genes [23–25].

The eusocial Hymenoptera (wasps, ants, and bees) occupy diverse thermal environments from low to high latitudes [26] and elevations [27, 28], suggesting that temperature may have played an important selective role in their evolution [29]. Species employ a variety of behavioral [30, 31] and physiological strategies [32] to reduce individual and colony-level exposure to thermal stress. However, individual foragers that leave the nest each day and immobile brood that develop in the nest are likely to encounter sufficiently high temperatures to trigger the HSR [33, 34]. Although key members of Hsp90 and Hsp70 have been identified in a few species of Hymenoptera [33–36], the diversity, functional properties, and regulation of molecular chaperones underlying adaptive variation in Hymenopteran thermal tolerance are poorly understood.

In this study, we evaluated the diversity and evolutionary history of Hsps across 12 species of Hymenoptera and five outgroup species (*Culex quinquefasciatus, Drosophila melanogaster, Bombyx mori*, *Tribolium castaneum*, *Acyrthosiphon pisum*) spanning four insect orders. We analyzed recently published genomes of multiple species of ants [37–42], bees (*Apis* [43] and *Bombus* [44]), and the jewel wasp (*Nasonia vitripennis* [45]) to identify orthologues within each major Hsp gene family and to characterize the upstream regulatory motifs governing their transcription (HSEs). We reconstructed molecular evolutionary relationships within each Hsp multigene family to identify evolutionary gains and losses and tested for positive or purifying selection for each homologous Hsp among lineages and across sites. To characterize the evolution of cis-regulation and identify Hsps involved in the HSR, we identified cis-regulatory HSEs within the promoter region for each homologous Hsp. We then tested whether HSE presence and configuration successfully predicted inducibility in two species of ants that experience different thermal environments: the hot-climate *Pogonomyrmex barbatus,* which inhabits deserts of the southwestern United States [46], and the cool-climate *Aphaenogaster picea*, which inhabits temperate deciduous forests of the eastern United States [47]. We found that ants, and probably other Hymenoptera, harbor unique, lineage-specific sets of heat inducible Hsps that were shaped by evolutionary gains, losses, and shifts in cis-regulation. Expression patterns of these heat-inducible Hsps reflect adaptive variation in thermal tolerance between *P. barbatus* and *A. picea*.

## Results

### Identification of conserved Hsp and cis-regulatory HSEs

We recovered conserved Hsps from all of the major gene families (Hsp90, Hsp70, Hsp60, Hsp40, small Hsps; Table 1). Three paralogues within the Hsp90 gene family (*trap1*, *gp93*, and *hsp83*) were found across all surveyed insects. We recovered five of the six *Drosophila melanogaster* Hsp70 homologues (CG2918, *hsc70-3* (BIP), *hsc70-4*, *hsc70-5*, and *hsp70CB*; Table 1) for Hymenoptera. With the exception of *Nasonia vitripennis*, the Hymenopteran taxa all lacked the heat-inducible orthologue *hsp70* (Table 1). For all species, we recovered two paralogues of Hsp60 (Table 1). Hsp40 gene families are one of the most diverse Hsps, but we narrowed our search to *DnaJ-1*, which is the known heat-inducible paralogue of *D. melanogaster* (Table 1). We did not recover a *DnaJ-1* paralogue from any of the insects surveyed and found the best BLAST match to be *D. melanogaster CG5001* (Table 1). Forward BLAST searching for *D. melanogaster* sHsps (*hsp22*, *hsp23*, *hsp26*, *hsp27*) yielded no reciprocal BLAST hits; instead, the closest match was *lethal 2 essential for life* (*l(2)efl)*, for which there were 3–9 copies in the Hymenoptera, and 1–17 copies in other members of the outgroup (Table 1).

**Table 1:** Summary of orthologous HSPs from the combination of reciprocal BLAST and HMMER searches using *D. melanogaster* as the reference

		Outgroup				Hymenoptera		
Gene family	Gene	*C. quinque.*	*T. castaneum*	*B. mori*	*A. pisum*	Ants	Bees	*N. vitripennis*
Hsp90	*trap1*	1	1	1	1	1	1	1
	*gp93*	3	1	1	1	1	1	1
	*hsp83*	3	3	1	2	1–2*	2	2
Hsp70	*CG2918*	1	1	1	3	1	1	1
	*hsc70-3 (BIP)*	1	1	1	1	1*	1	1
	*hsc70-4*	2	1	1	2	2*	2	2
	*hsc70-5*	1	1	1	1	1*	1	1
	*hsp70*	6	1	2	3	0	0	1
	*hsp70CB*	1	1	1	1	1	1	1
Hsp60	*tcp-1*	1	1	1	1	1	1	1
	*hsp60*	1	1	1	1	1*	1	1
Hsp40	*CG5001*	1	1	1	1	1*	1	1
small Hsps	*hsp23*	0	0	0	0	0	0	0
	*hsp24*	0	0	0	0	0	0	0
	*hsp26*	0	0	0	0	0	0	0
	*hsp27*	0	0	0	0	0	0	0
	*l(2)efl*	8	10	17	1	3–6*	4–9	7

Each entry is the number of orthologous HSPs detected. The astericks (*) indicate orthologues that were detectable by qPCR. For *l(2)efl*, only one paralogue was detectable by qPCR. *C. quinque* = *Culex quinquefasciatus, T. castaneum* = *Tribolium castaneum. B. mori* = *Bombyx mori, A. pisum* = *Acyrthosiphon pisum, N. vitripennis* = *Nasonia vitripennis*. See text for further details of ants and bees used for analysis

**Fig. 1:**
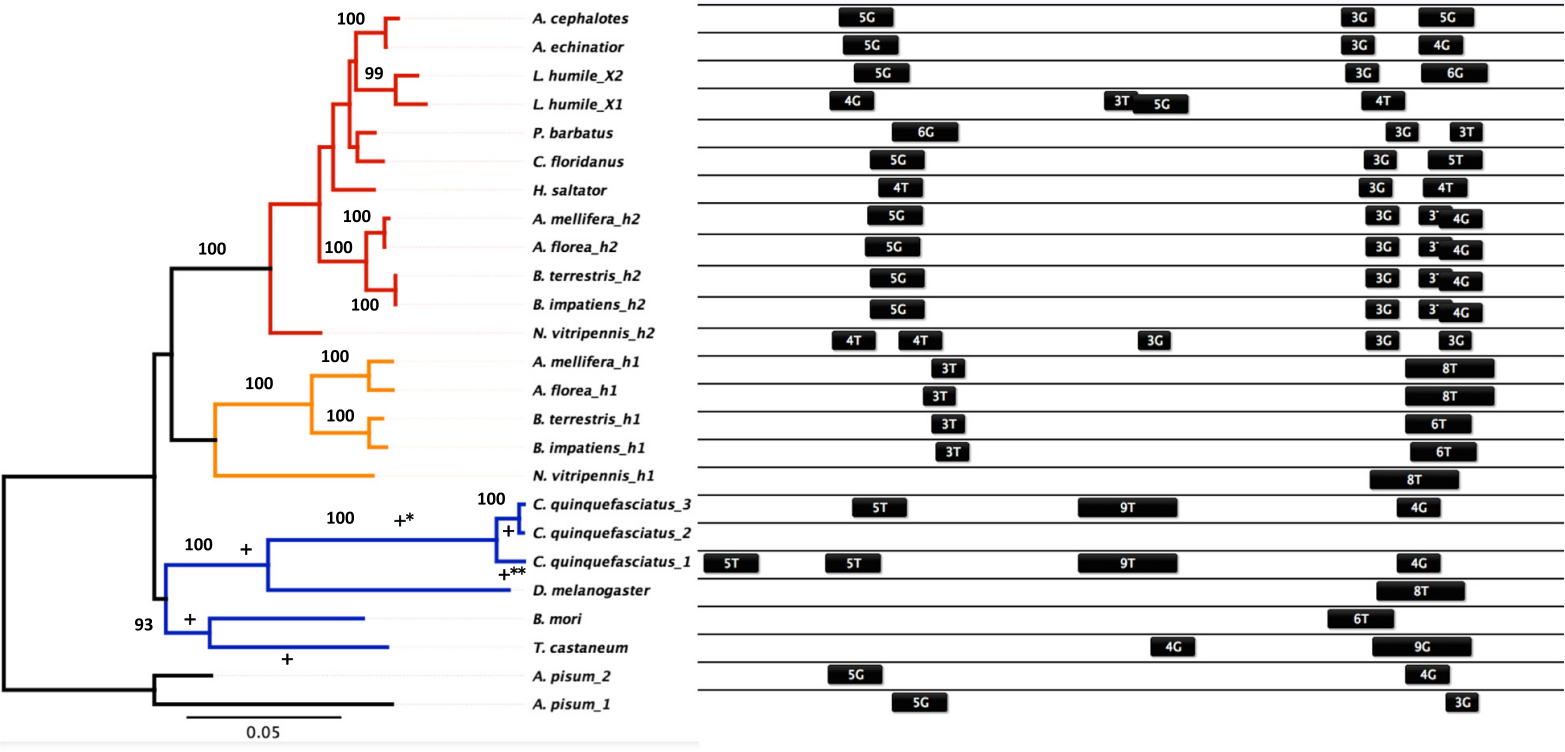
Evolutionary gains and losses in *hsp83* within Hymenoptera, followed by diversification in cis-regulatory HSEs. Relationships of homologous *hsp83* were reconstructed with PhyML for 17 insect species (rooted with *A. pisum*) using a JTT substitution model with 1000 bootstrap replicates (>90 bootstrap support indicated; left). Branches of the outgroup taxa are colored in blue and black, while well-supported paralogues of Hymenopteran branches are colored in orange (h1) and red (h2). Statistically significant episodic diversifying selection using Branch-Rel is indicated along the branch (+ corresponds to *p* < 0.05; * = *p* < 0.01; ** = *p* < 0.001). Cis-regulatory HSEs in the promoter region spanning 400 bps from the transcription start site (TSS; right) are mapped onto the phylogeny and are annotated by their length and motif type

**Fig. 2:**
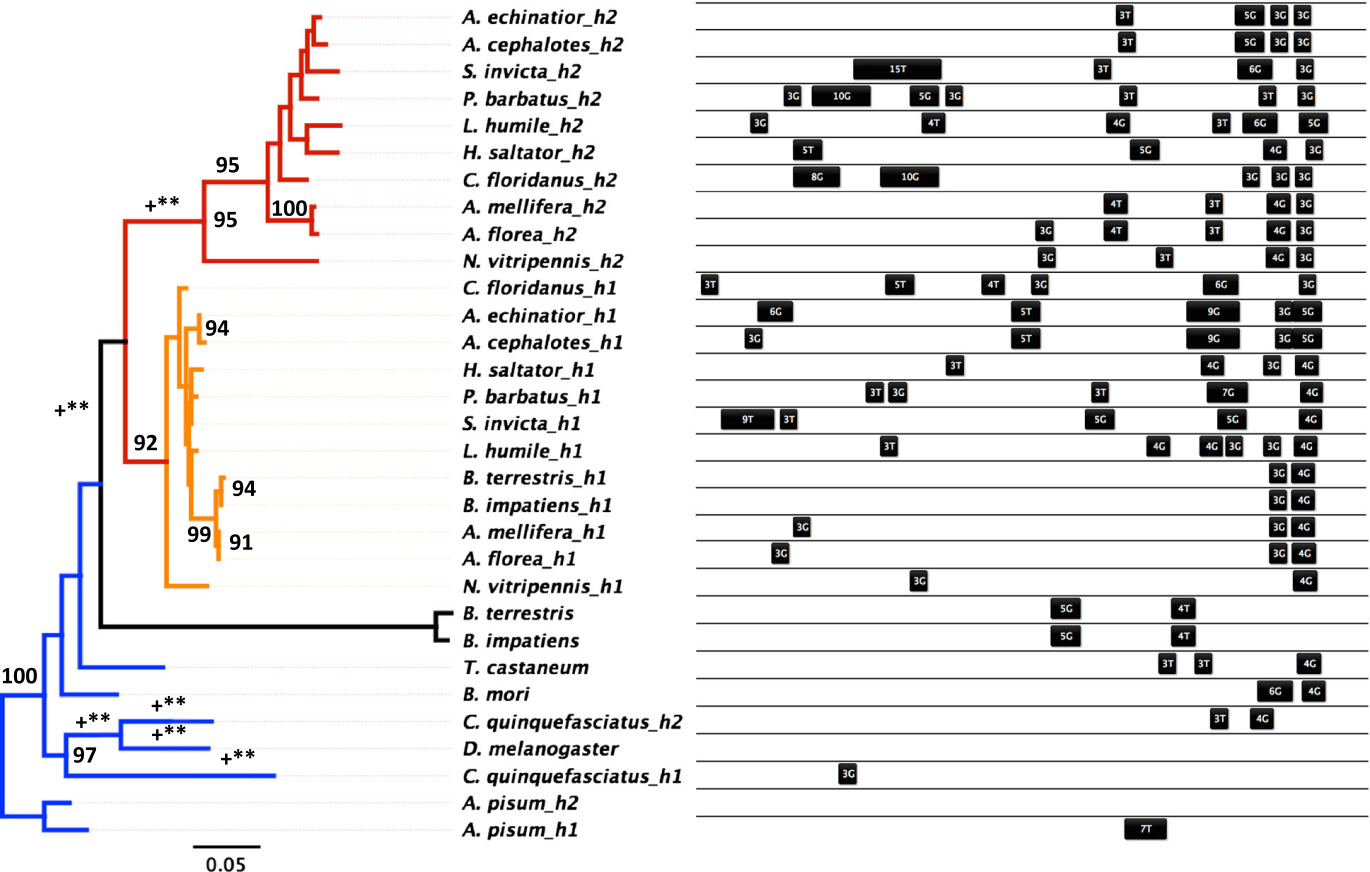
Evolutionary conservation of two copies of *hsc70-4* within Hymenoptera, but both copies harbor an extraordinary amount of diversity in cis-regulatory HSEs. Relationships of homologous *hsc70-4* were reconstructed with PhyML for 17 insect species (rooted on *A. pisum*) using a JTT substitution model with 1000 bootstrap replicates (>90 bootstrap support indicated; left). Branches of the outgroup taxa are colored in blue, while well-supported paralogues of Hymenopteran branches are colored in orange (h1) and red (h2). Statistically significant episodes of positive selection identified with Branch-Rel are indicated along the branch(+ corresponds to *p* < 0.05; * = *p* < 0.01; ** = *p* < 0.001). Cis-regulatory HSE elements in the promoter region spanning 570 bps from the transcription start site (TSS; right side) are mapped onto the phylogeny and are annotated by their length and motif type

**Fig. 3:**
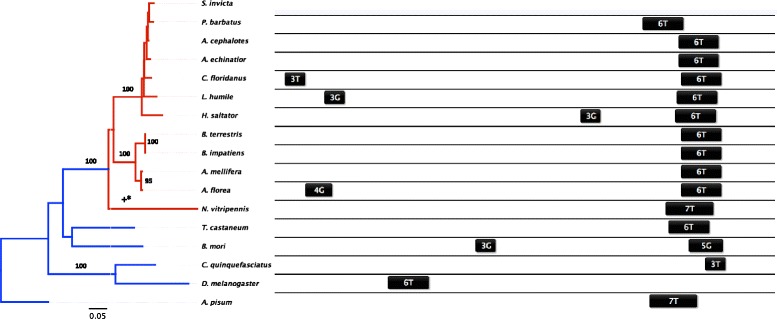
Evolutionary conservation of *hsp40* copy number and cis-regulatory HSEs. Relationships of homologous hsp40 were reconstructed with PhyML for 17 insect species (rooted on A. pisum) using a JTT substitution model with 1000 bootstrap replicates (>90 support indicated). The outgroup and Hymenopteran branches are indicated in blue and red, respectively. Statistically significant episodes of positive selection using Branch-Rel are indicated along the branch (+ corresponds to *p* < 0.05; * = *p* < 0.01; ** = *p* < 0.001). Cis-regulatory HSE elements in the promoter region spanning 370 bps from the transcription start site (TSS; right side) are mapped onto the phylogeny and are annotated by their length and motif type. *S. invicta* did not provide enough sequence information for the identification of cis-regulatory HSEs

Of the Hsp homologues, eight were quantifiable by qPCR and were subsequently searched for cis-regulatory HSEs (Table 1, indicated with asterisks). Local alignment of the promoter regions of *hsp83*, *hsc70-4* (h1 and h2), and *hsp40* across species indicated conserved location, conformation, and arrangement of cis-regulatory HSEs (Figs. 1, 2 and 3), whereas *hsc70-3* (BIP), *hsc70-5*, *hsp60,* and *l(2)efl* had less conserved HSEs (Additional file 1: Figures S1, Additional file 2: Figures S2, Additional file 3: Figures S3; data not shown for *l(2)efl*). For Hsps with conserved HSEs, 193 HSE motifs were annotated, including 114 head types (‘nGAAn’) and 79 tail types (‘nTTCn’; Figs. 1, 2 and 3). Across all sampled insects, we found no consistent preference for head or tail motifs in *hsp83* (exact binomial test, *p* = 0.055), significant preference for the head motif in *hsc70-4* (*p* < 0.001), and significant preference for the tail motif in *hsp40* (*p* < 0.05).

### Heat shock protein (Hsp) and cis-regulatory heat shock element (HSE) evolution

#### Hsp83

Phylogenetic reconstruction of *hsp83* revealed multiple duplications and losses in both the outgroup and Hymenoptera (Fig. 1). An early duplication event in a common ancestor of the Hymenoptera generated two paralogues of *hsp83* (h1 and h2 in Fig. 1). Although both paralogues are present in bees and wasps, only one paralogue (h2) exists in ants, indicating a secondary loss. A second duplication of the h2 orthologue occurred in *Linepithema humile.* Selection analysis along the length of the gene sequence indicated that most sites (608/714 and 625/714, Single likelihood ancestor counting (SLAC) and Relative effects likelihood (REL) analyses, respectively, Table 2) identified purifying selection; there was no evidence for episodic diversifying selection in branches leading to Hymenopteran paralogues (Branch-REL, *p* > 0.5; Fig. 1).

**Table 2:** Summary of selection analyses for three HSP genes

			Global ω		ω-/ω+		
Gene	N	Codons	SLAC	REL	SLAC	FEL	MEME
*hsp83*	25	714	0.0603	0.071	608/0	625/0	NA/1
*hsc70-4*	31	710	0.0549	0.051	608/0	610/0	NA/7
*hsp40*	17	384	0.1147	0.100	253/0	284/0	NA/1

For each gene, the number of sequences and number of codons were used for detecting positive selection. The mean global ω is shown for SLAC and REL methods. The number of sites that are negatively or positively selected are shown under ω -/ ω + for SLAC, FEL. *P*-values were set to default (*p* = 0.1) for SLAC, FEL, REL. MEME provides evidence for episodic positive selection at individual branches and sites (*p* < 0.01) and the number of negatively selected sites are non applicable (NA)

In spite of overall sequence conservation, Hymenopteran *hsp83* h2 differs in genomic structure and cis-regulation from Hymenopteran *hsp83* h1 and from outgroup species in three ways. First, Hymenopteran *hsp83* h1 and most outgroup species completely lack introns, whereas *hsp83* h2 has two introns; *Apis mellifera hsp83* h1 is the exception, with one intron in *hsp83* h1 (Additional file 4: Figure S4). Second, Hymenopteran *hsp83* h2 has a split HSE arrangement (4–6 and 3 HSE motifs), whereas both *hsp83* Hymenopteran h1 and the outgroup have a contiguous HSE arrangement (6–9 HSE motif length) at the proximal end of the molecule (30–100 bps upstream TSS; Fig. 1). Third, there is a preference in head-type motifs only in Hymenopteran *hsp83* h2 (Fisher’s Exact Test, *p* <0.001; Fig. 1).

#### Hsc70-4

Phylogenetic reconstruction of *hsc70-4* indicates multiple duplication events both within species (*C. quinquefasciatus* and *A. pisum*) and in a common ancestor of the Hymenoptera, leading to two paralogues (h1 and h2; Fig. 2). Each paralogue forms a strongly supported clade, with the exception of the two *Bombus* species, in which the h1 paralogue is nested within the h1 clade but the second copy does not group with either Hymenopteran paralogue (Fig. 2). There is evidence of episodic diversifying selection along the branch preceding the *hsc70-4* duplication in the Hymenoptera and also in the Hymenopteran *hsc70-4* h2 lineage (Branch-REL, *p* <0.001 in both cases; Fig. 2), even though most individual sites (608/710 and 610/710, SLAC and REL analyses, respectively) were under purifying selection (Table 2).

Hymenopteran *hsc70-4* differs in genomic structure and cis-regulatory HSEs from that of *D. melanogaster*. The orthologue of *hsc70-4* in *D. melanogaster* lacks introns and cis-regulatory HSEs (Additional file 5: Figure S5; Fig. 2). In contrast, Hymenopteran *hsc70-4* h1 has one intron, with the exception of *N. vitripennis,* which has two introns. Hymenopteran *hsc70-4* h2 also has two introns, with the exception of *Bombus* (Additional file 5: Figure S5). Compared to the *hsc70-4* in members of the outgroup (Fig. 2, *right*), both Hymenopteran *hsc70-4* paralogues showed high diversification in cis-regulatory HSEs, particularly at the more distal positions ( >120 bps upstream TSS). At the proximal position (30–115 bps upstream TSS), however, HSEs of Hymenopteran *hsc70-4* aligned locally with the inducible *D. melanogaster hsp70* gene (data not shown).

#### Hsp40

Both sequence and copy number of *hsp40* were phylogenetically conserved across all insect species (Fig. 3). Most sites were under purifying selection (Table 2), and there was no evidence of episodic diversifying selection along branches leading to the Hymenoptera (Fig. 3). Cis-regulatory HSEs of *hsp40* were concentrated in one conserved proximal block of 3–7 HSE subunits that were located 35–100 bps upstream of the TSS, although in *D. melanogaster* HSEs were located 255–285 bps upstream (Fig. 3). However, the genetic structure appears less conserved, ranging from zero to three introns (Additional file 6: Figure S6).

### Inducible Hsp expression

**Fig. 4:**
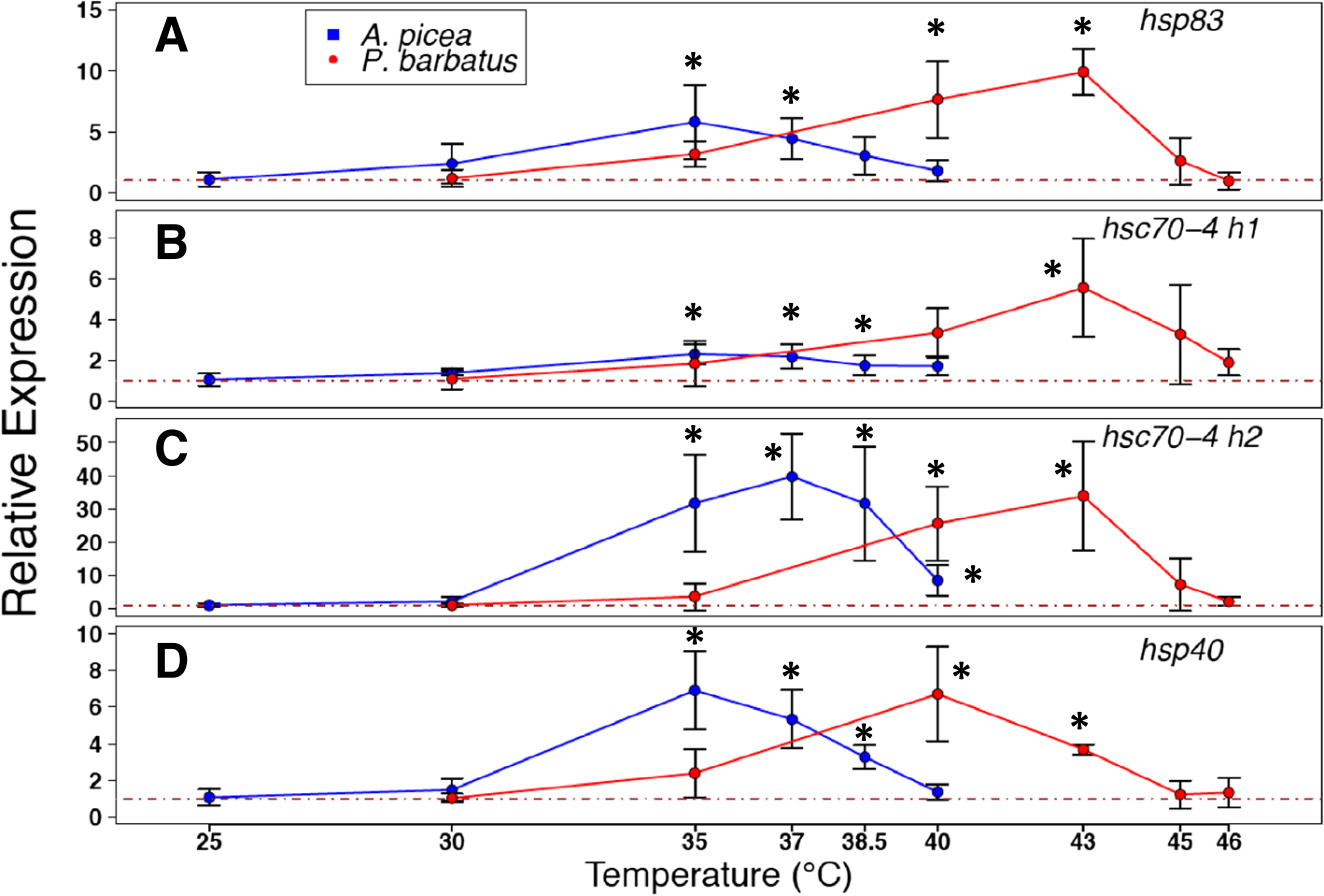
Relative fold increase in gene expression (+/− SD) for four inducible HSPs in *A. picea* and *P. barbatus* across different temperature treatment. Relative expression of *hsp83* (**a**), *hsc70-4* h1 (**b**), *hsc70-4* h2 (**c**), and *hsp40* (**d**) were normalized to the 18 s rRNA and β-actin, 18 s rRNA and *GAPDH* in *A. picea* (*N* = 4 per treatment) and *P. barbatus* (*N* = 3 per treatment), respectively. Significant up-regulation from 25 °C (*A. picea*) and 30 °C (*P. barbatus*) is denoted by ‘*’ from *post hoc* Tukey tests (*p* < 0.05)

**Fig. 5:**
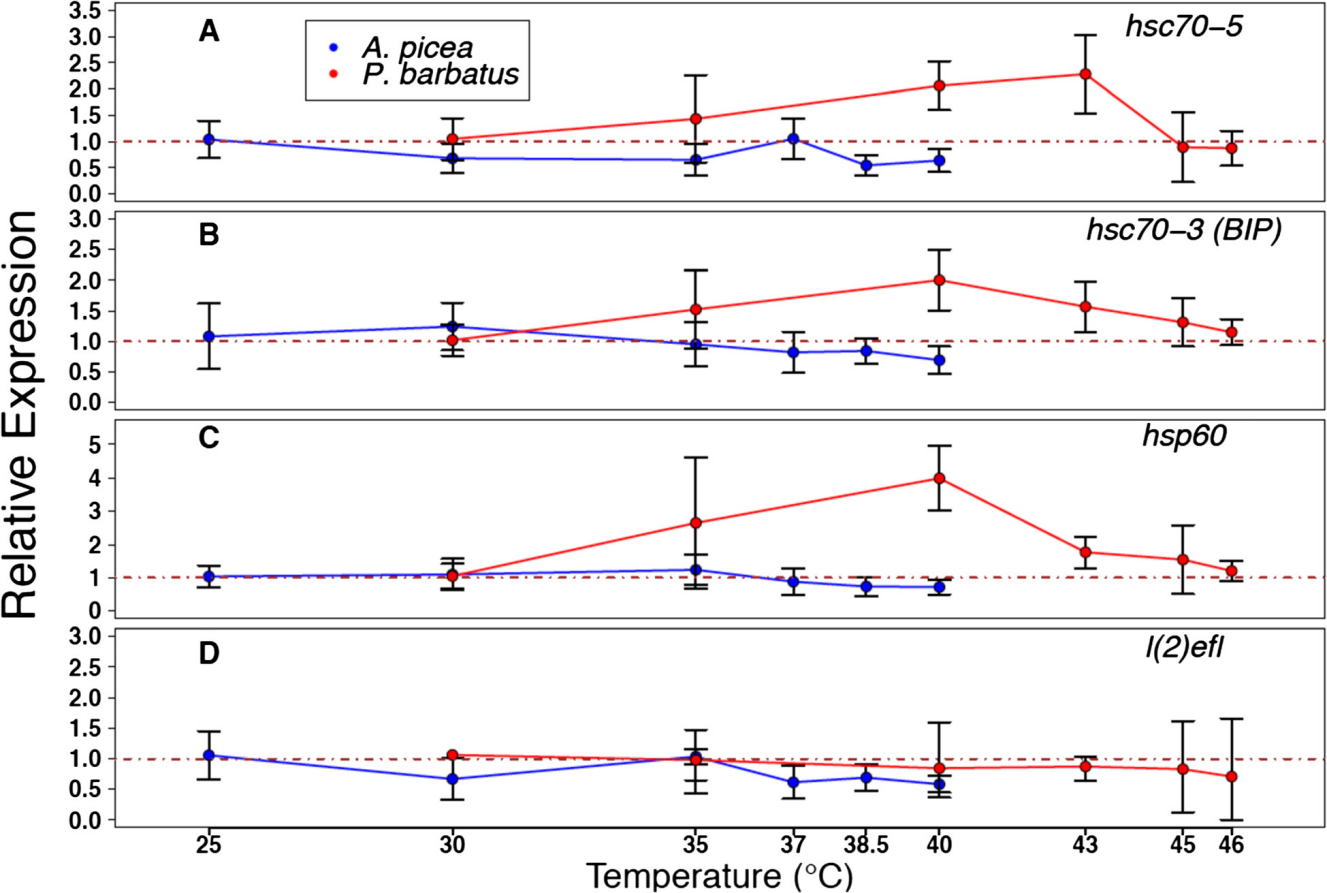
Relative fold change in gene expression (+/− SD) for four non-inducible Hsps in *A. picea* and *P. barbatus* across different temperature treatment. Relative expression of *hsc70-5* (**a**), *hsc70-3* (**b**), *hsp60* (**c**), and *l(2)efl* (**d**) were normalized to the 18 s rRNA and β-actin and 18 s rRNA and GAPDH for *A. picea* (*N* = 4 per treatment) and *P. barbatus* (*N* = 3 per treatment), respectively

We tested whether the presence or absence of conserved cis-regulatory HSEs successfully predicted Hsp gene induction in response to experimental heat shock. The four Hsp genes with conserved HSEs were all significantly up-regulated in response to increasing temperature treatments (*hsp83* (F_5,12_ = 8.48; *p* < 0.01), *hsc70-4* h1 (F_5,12_ = 3.74; *p* < 0.05), *hsc70-4* h2 (F_5,12_ = 10.6; *p* < 0.001), and *hsp40* (F_5,12_ = 6.97, *p* < 0.01); Fig. 4a–d). The other four Hsps, which lacked conserved HSEs, were not significantly up-regulated after heat shock (*hsc70-5* (F_5,12_ = 2.17; *p* = 0.13), *hsc70-3* (F_5,12_ = 1.91; *p* = 0.17), *hsp60* (F_5,12_ = 2.86; *p* = 0.063), and *l(2)efl* (F_5,12_ = 0.223; *p* = 0.946); Fig. 5a–d).

### Species comparisons

**Fig. 6:**
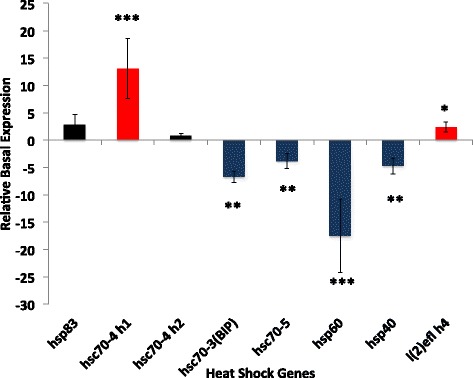
Relative basal heat shock gene (target) expression (+/− SD) between *P. barbatus*(*N* = 3) and *A. picea* (*N* = 4). Relative gene expression was normalized with the geometric mean of 18 s rRNA and β-actin as the calibrator (* = *p* < 0.05;** = *p* < 0.01; *** = *p* < 0.001 levels of significance) and fold change was calculated as *P. barbatus* relative to *A. picea* was calculated as follows: 2^Target(Pbar-Apic)^/2^Calibrator(Pbar-Apic)^(Pbar = *P. barbatus*, Apic = *A. picea*). -1 was divided by values less than one to calculate negative relative basal expression. Significant up-regulation in *P. barbatus* and *A. picea* are colored in red and blue, respectively

**Fig. 7:**
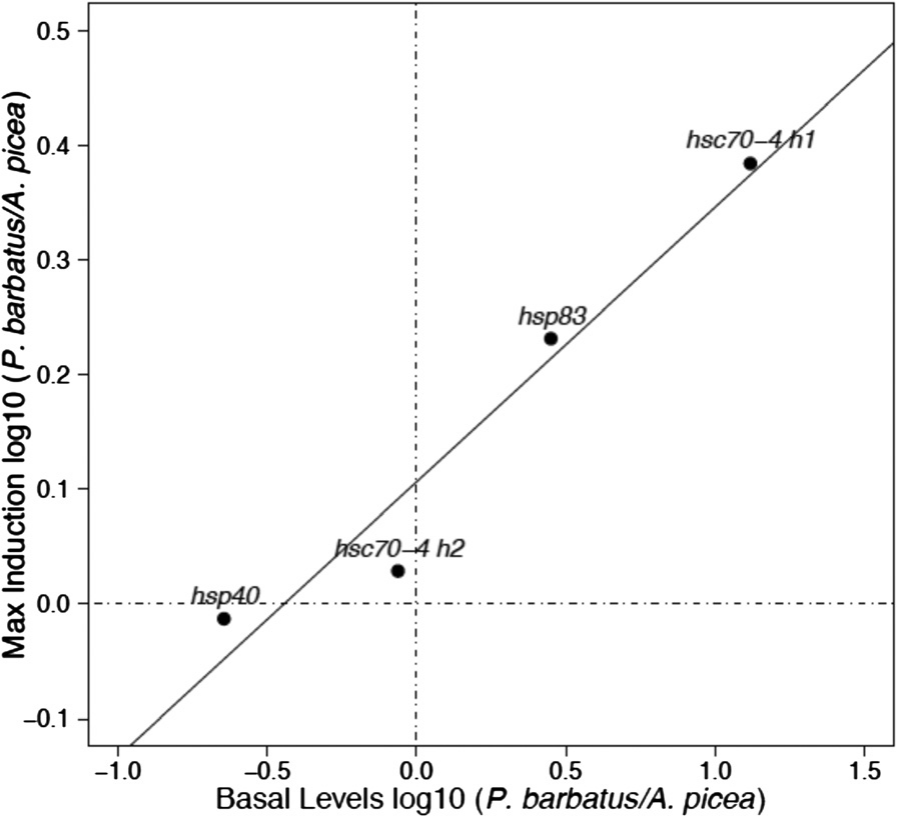
The positive relationship between the log ratios of basal expression levels (*P. barbatus*/*A. picea*) at rearing temperatures and max induction (β_1_ slope = 0.2398, r^2^ = 0.918, *p* < 0.05)

We then tested whether variation in thermal tolerances between two ant species was accompanied by changes in Hsp inducibility. The median lethal temperature 50 (LT_50_) of the warm-climate *P. barbatus* (median LT_50_ = 46.9 **°**C) was significantly higher than the LT50 of the cool-climate *A. picea* (median LT_50_ = 38.78 °C; generalized linear model (GLM) with a binomial response variable: influence of species, *p* < 0.001; Additional file 7: Figure S7). These survivorship differences were matched by patterns of Hsp gene expression: *P. barbatus* shifted its expression profile toward higher temperatures than did *A. picea* for all inducible Hsps (Fig. 4a–d). For *hsp83*, *hsc70-4 h1*, and *hsc70-4* h2, *P. barbatus* showed peak expression at 43 °C, whereas *A. picea* showed peak expression at 35–38.5 °C (Fig. 4a–c). For *hsp40*, peak expression was 40 and 35 °C for *P. barbatus* and *A. picea*, respectively (Fig. 4d). *P. barbatus* exhibited significantly higher constitutive expression of *hsc70-4* h1 (ANOVA, F_1,5_ = 87.8, *p* < 0.01) and *l(2)efl* (F_1,5_ = 6.92, *p* < 0.05), and significantly lower constitutive expression of *hsc70-3* (F_1,5_ = 596, *p* < 0.01), *hsc70-5* (F_1,5_ = 24.3, *p* < 0.001), and *hsp60* (F_1,5_ = 31.2, *p* < 0.01) than did *A. picea* (Fig. 6). Among the inducible Hsps, there was a positive relationship between relative basal expression levels and relative inducibility (linear regression, r^2^ = 0.918, *p* < 0.05; Fig. 7).

## Discussion

Molecular characterization of Hymenopteran Hsps reveals functionally important divergence in identity, amino acid sequence, and regulation of chaperone proteins (Table 2, Figs. 1 and 2). Both *hsp83* and *hsc70-4* display Hymenoptera-specific gains and losses, resulting in unique sets of homologues. Although most codons exhibited purifying selection (Table 1), instances of positive selection along branches leading to and within the Hymenoptera (Figs. 1 and 2, left) suggest novel chaperoning activity [48]. This sequence divergence, coupled with cis-regulatory HSE distribution and expression patterns (Figs. 1 and 2, right; Figs. 4 and 5), suggests that although there is substantial conservation of ancestral inducibility, the HSR response in Hymenoptera has been additionally augmented by expansion and subfunctionalization of novel gene duplicates that are activated by thermal stress.

As in other taxonomic groups, cytoplasmic Hsps mainly mediate the HSR in Hymenoptera (Figs. 4 and 5), whereas mitochondrial and ER-localizing forms of Hsp70 [9, 49] and Hsp90 appear to play little role ([50, 51], but see [52]). The set of inducible Hsps identified likely interact with one another to protect and refold denatured proteins. Upon protein denaturation, Hsp40 delivers unfolded proteins to Hsp70, and the two together mediate refolding through cycles of substrate binding and release driven by ATP binding and hydrolysis [53]. Despite their interdependence, however, the extent of functional diversification of *hsc70-4* and *hsp40* differed substantially (Figs. 1, 2 and 3). *Hsc70-4* showed the most dramatic variation, with the primary inducible member *hsp70* in *Drosophila* completely lost in Hymenoptera, which instead induces two *hsc70-4* paralogues that vary in both HSE configuration and fold-increase in response to heat stress (Figs. 2 and 4). Utilization of *hsc70* in the stress response across the insects appears to be widespread, with HSEs present in most of the taxa sampled (Fig. 2). *Hsc70-4* also contains gene duplications in other taxa, suggesting that this gene family has undergone multiple evolutionary gains, losses and functional shifts. For example, *Culex quinquefasciatus* has two paralogues, one of which is accompanied by cis-regulatory HSEs (Fig. 2), suggesting that one copy is heat-inducible and the other serves housekeeping functions.

For *hsp83*, we found two paralogues in bees and wasps, one with an ancestral contiguous arrangement of HSEs, and one with a derived split arrangement similar to that of *Drosophila hsp70*. This split arrangement reduces cooperative binding of HSF trimers, leading to lower basal expression and higher inducibility than in the more contiguous motif [14, 54, 55]. The presence of two differentially regulated paralogues may reflect novel functionalization in *hsp83* to provide both basal and inducible Hsp expression. Foraging bees are known to super-heat thoracic muscles prior and during flight, which necessitates both constitutive and inducible chaperoning activity [32, 34]. Transcriptomic screens in *Apis mellifera* have found weak support for Hsp90 up-regulation in foraging relative to nurse bees, but more detailed and precise quantification of each paralogue will determine whether they have subfunctionalized into constitutive and inducible roles [56]. In contrast, except for the nuptial flight of males and queens, worker ants are flightless, which may explain the secondary loss of the ancestral paralogue but the retention of the more inducible form.

In contrast to *hsc70* and *hsp83, hsp40* was much more conserved. There was a single gene copy per taxon in which most sites were under purifying selection, suggesting that their co-chaperoning activity has been retained across insects. In particular, the conserved J domain of Hsp40 stimulates the ATPase domain of Hsp70 proteins. Across the insect *hsp40* phylogeny, HSE configuration remained conserved for all but *D. melanogaster*, whose primary motif was further from the transcriptional start site (Fig. 3). Although the Hsp40 gene family is one of the most diverse molecular chaperones, we captured the paralogue that participates in the HSR because it was significantly up-regulated in response to heat stress. Interestingly, *hsp40* in *P. barbatus* peaked in up-regulation at a less extreme temperature than did the other Hsp proteins (Fig. 4d). Early expression of *hsp40* may enhance chaperoning activity by binding to existing and accumulating pools of *hsc70* and also by providing crosstalk with Hsp90-mediated chaperoning [57].

Comparisons of two ant species that experience very different thermal ranges revealed correlated shifts in both the basal expression and inducibility of Hsps that reflect the higher and more frequent thermal stress expected in extreme habitats (Fig. 7, Additional file 7: Figure S7). Workers of the harvester ant *P. barbatus* forage in extreme desert heat [58, 59] and may be more reliant on both constitutive and inducible mechanisms to cope with thermal stress than workers of *A. picea*, which are more temperature sensitive and occur in thermally buffered mesic deciduous forest [47, 60]. The gene expression responses of *P. barbatus* and *A. picea* are consistent with previous work comparing two hot-desert ant species of *Cataglyphis* with the cool woodland ant *Formica polyctena* [33]. In that study, HSP70 (*hsc70-4*) basal expression and induction were higher in *Cataglyphis*, although alternative paralogues were not fully distinguished. Although Hsp chaperoning activity expends energy (ATP), there may not be trade-offs between continual and maximum induction of Hsps because investment in the HSR is less costly than the loss of biochemical activity from protein denaturation [4, 61, 62]. In addition, the HSR in *P. barbatus* in this study was shifted upward by ~5–7 °C (Fig. 4), suggesting underlying differences in overall proteome stability that permit *P. barbatus* to tolerate significantly higher temperatures than *A. picea* (Additional file 7: Figure S7).

## Conclusions

Our study represents the most comprehensive survey to date of Hsp sequence and cis-regulatory evolution for insects. Hymenoptera have unique Hsp evolutionary histories shaped by gains, losses, and changes in cis-regulation. Based on the presence of conserved cis-regulatory elements (HSEs), we reliably predicted the heat inducible Hsps that are critical for mounting the HSR in ants, suggesting that the ancestral inducibility has been retained. We uncovered greater diversity in the number, arrangement, and location of cis-regulatory HSEs in Hymenoptera for two major Hsp genes (*hsp83* and *hsc70-4*), suggesting that the HSR is mediated through changes in cis-regulation. Furthermore, Hsp expression patterns were associated with the thermal limits of two ant species that inhabit different thermal environments. Collectively, our analyses suggest Hsp sequence and expression patterns may reflect the forces of selection acting on thermal tolerance in ants and other social Hymenoptera.

## Methods

### Phylogenetic reconstruction

To reconstruct the evolutionary relationships of heat shock proteins, we identified orthologous Hsps in 17 insect species representing five insect orders using the well-characterized Hsps of *Drosophila melanogaster* as a reference (Additional file 8: Table S1). Reciprocal best BLAST (blastp) searches (e-value < 1E-10, and top bit score) were used to identify annotated orthologues of the known *D. melanogaster* paralogues with an ant-specific genome database (http://antgenomes.org/, [63]) as well as with the NCBI non-redundant protein and nucleotide databases (Additional file 8: Table S1). To find unannotated sequences, we queried *D. melanogaster* orthologues with tblastn to each insect species’ genome. To identify additional homologues not found with BLAST, we employed a similar search with Hmmer 3.0 [64]. We used *Drosophila melanogaster* transcripts to search (*hmmsearch*) each individual genome and identified orthologues based on e-value < 1E-10 and top bit score. HMMER searches recovered nine additional copies from two genes (*gp93* and *hsp70*) for *Culex quinquefasciatus*. Identified nucleotide sequences were translation-aligned with MAFFT using default settings [65] to identify homologous codons for subsequent selection analyses and the resultant alignment was translated for phylogenetic reconstruction [66]. We reconstructed gene relationships of homologous Hsps with PhyML [66, 67], and bootstrap support was estimated for 1000 replicate searches utilizing an amino acid substitution model inferred from Prottest3 [68]. Similar phylogenetic relationships were recovered with a Bayesian analysis using MrBayes [66, 69].

### Tests of selection

Selection at the protein-coding level was quantified as the ratio of the nonsynonymous substitution rate to the synonymous substitution rate (ω = d_N_/d_S_); ω > 1 indicates positive selection, whereas ω < 1 indicates purifying selection, and ω = 1 is indicates neutral evolution [70]. For each homologous Hsp, we tested for selection at individual codons as well as across the phylogeny using the HyPhy package [71] on the web-interface Datamonkey (http://www.datamonkey.org).

We identified individual codon sites for positive selection using Single-Likelihood Ancestor Counting (SLAC), Random Effects Likelihood (REL), and Fixed Effects Likelihood [72]. SLAC calculates the number of observed and expected d_N_ and d_S_ rates and conservatively estimates ω using a recommended cutoff of *p* = 0.1 [72]. The REL method is an extension of analyses in PAML [70] that allows d_N_ and d_S_ to vary across sites and uses a Bayes factor (>50) to assess selection [72]. FEL estimates d_N_ and d_S_ from the codon substitution model and implements a likelihood ratio to evaluate significance using a recommended cutoff of *p* = 0.1 [72].

In addition to testing for selection at sites along the gene, we tested for changes in selective pressures across the reconstructed amino acid phylogeny, which might indicate evolutionary shifts in gene function. Episodic diversifying selection was assessed using branch-REL and MEME [73, 74]: branch-site REL detects episodic diversifying selection for individual lineages [73], whereas MEME is an extension of FEL that detects episodic diversifying evolution by allowing ω to vary across branches and sites [74].

### Identification of genomic structure and cis-regulatory Heat Shock Elements (HSE)

Identification of genomic structure and cis-regulatory HSEs was performed for Hsps that were detectable by qPCR (for methods, see Quantitative real time PCR). We mapped transcripts to their respective genomic regions in Geneious Pro 6.1 [75] and identified exons and introns, making further manual alignments by hand when necessary. The transcriptional start site (TSS) was predicted using Neural Network Promoter Predictor (NNPP) [76]. Previous chip-seq experiments in *D. melanogaster* revealed that HSF binds primarily to Hsp promoters within 1250 bps of the TSS [77]; sequences were trimmed to this length and locally aligned to identify orthologous HSEs.

To identify cis-regulatory HSEs, we followed a modified search procedure adapted from Tian et al. [17]. Promoter sequences were searched for the putative HSE motif (head conformation = nGAAnnTTCnnGAAn or tail conformation = nTTCnnGAAnnTTCn) [78], allowing for a two base-pair mismatch from the preferred sequence [66]. HSE motifs were then manually screened and annotated by the number and type of subunit occupying the distal end (subunits beginning with ‘nGAAn’ or ‘nTTCn’ refer to the head or tail conformation, respectively). Mismatches occurring at critical sites for HSF binding (G in 2nd position of head conformation, C in 4th position of tail conformation) [79] were discarded, unless motifs were interior to a HSE with three or more subunits, known as a gapped HSE [17].

A final screen was employed to quantify the binding strength of each HSE subunit. Briefly, a WebLogos [80] was generated for head and tail types recovered from the search. Bit scores for the preferred base at each of the five possible positions in a subunit were summed; the match between the individual subunits and the preferred subunit was expressed as the ratio of the summed observed bit score over the preferred bit score. Subunits with scores less than 0.5 were discarded unless flanked with subunits with scores greater than 0.5, again indicating a ‘gapped’ HSE. 253 out of 1753 total HSEs were retained after screening (Additional file 9: Table S2).

### Field collections and lab rearing

Hsp induction was quantified in workers sampled from lab-acclimated colonies of *Pogonomyrmex barbatus* and *Aphaenogaster picea*. Three *Pogonomyrmex barbatus* colonies were reared from queens collected with permission following a mating flight at the Welder Wildlife Foundation in Sinton Co., Texas (28.10837 °N 97.42265 °W) in June 2007. Colonies were maintained in an environmental room at the University of Vermont, Department of Biology, with a 12:12 light dark light cycle at 30 **°**C in 17 × 12 × 6 cm plastic nest boxes provided with three 16 × 150 mm disposable glass test tubes in which water was supplied behind a cotton stopper as a nest site. Each week, colonies were fed two mealworms (*Tribolium molitor*) and an *ad libitum* seed mixture composed of oat bran, wheat germ, millet, thistle seeds, and quinoa.

Eight colonies of *A. picea* were collected with permission from the University of Vermont in May and June 2012 from black spruce forest adjacent to Molly Bog (44.508611°N, 72.702222°W), located near Stowe, Vermont. Entire live colonies containing 500–1000 workers, brood, and queen were excavated from the leaf litter. Colonies were maintained for 1 month in the laboratory at 25 °C +/− 1 °C with 12 h light/dark cycles in a 7 x 3 ¼ x 1 ¾ inch plastic nest box covered with red cellophane and connected to an open plastic foraging arena filled with ~1 cm sand and lined with Insect-a-slip (BioQuip) to prevent escape. 1–3 cotton-plugged water tubes (16 × 150 mm) were provided in the nest box for each colony to maintain humidity. Approximately 200 μl of 20 % honey water and one bisected mealworm were provided in each foraging arena every 3 days.

### Thermal tolerance assays

Acute upper thermal limits in both species were determined by quantifying a LT_50_ temperature, defined as the temperature at which a one-hour exposure produced 50 % worker mortality after 3 days of recovery using the *dose.p* function in the MASS package within R (version 3.2.0) [81]. Ants were exposed to six different temperature regimes (30, 35, 40, , 42, 45, 46 **°**C for *P. barbatus* and 25, 30, 35, 36.5, 38.5 40 **°**C for *A. picea*). Temperature treatments were applied by confining 10–13 nest-mate workers together in a 5 mL screw-cap glass vial and submerging the vial in a pre-set Thermo Neslab EX17 heating water bath for 1 h. Temperature inside the vials was monitored with a temperature probe inserted in an empty 5 mL glass vial submerged in the water bath simultaneously. After the application of temperature treatment, ten ants were allowed to recover for survival counts in a 16 × 150 mm cotton-plugged water tube. For each treatment, three ants per colony from four of the eight *A. picea* colonies and the three *P. barbatus* colonies were flash frozen and stored at −80 **°**C for gene expression analyses.

### Quantitative real time PCR

RNA was isolated from flash-frozen ants with RNAzol (Molecular Research Center, Inc., USA) and then purified with the RNeazy micro kit (QIAGEN, USA) for downstream gene expression quantification. Flash-frozen ants from each temperature treatment were pooled and homogenized in a Bullet Blender (Next Advance Inc., USA) homogenizer at top speed (10) with 1.4 mm zirconium silicate grinding beads (Quackenbush Co., Inc., USA) and 500 uL of RNAzol buffer (Molecular Research Center, Inc., USA) for 3 min. Following the manufacturer’s instructions for RNAzol, RNA samples were resuspended in 100 uL of water and subsequently purified with Rneasy micro kit with DNAse I (Qiagen, USA) treatment on the micro column to remove genomic DNA contamination. RNA was quantified with Nanodrop spectrophotometry; all sample 260/280 ratios were between 2.0–2.2, indicating acceptable RNA quality. mRNA was reverse transcribed into cDNA with High Capacity cDNA Reverse Transcription Kit (ABI, USA).

To detect specific heat shock proteins, primers were designed for a whole suite of genes for each gene family (Table 1, Additional file 10: Table S3). Table 1 highlights (in *) working primer sets. Quantitative PCR was performed on an ABI StepOnePlus Real-Time PCR system. Reactions were performed in 20 μl volumes with 2 ng of template cDNA, 500 nM total primer, and 10 μl of Power SYBR® Green Master Mix (Life Technologies, USA). Cycling conditions consisted of an initial 95 **°**C incubation for 2 min and then 40 cycles of 95 **°**C for 15 s, with 55 **°**C annealing and extension for 60 s. Following amplification, melt curve analyses confirmed the presence of a single amplicon. All gene products from preliminary experiments were sequenced for verification of specific gene amplification.

Gene expression fold changes were calculated relative to rearing temperatures using the ΔΔ CT method [82] after empirically determining ~100 % primer efficiencies for each primer set (Additional file 10: Table S3). The set of housekeeping genes for normalization was determined with Normfinder [83], which calculated the relative stability of four housekeeping genes (18 s rRNA, GAPDH, β-actin, and Ef1β) and selected the most stable genes across samples. For *A. picea*, 18 s rRNA and β-actin were most stable (0.20 stability), whereas 18 s rRNA and GAPDH (0.25 stability) were the most stable for *P. barbatus*. For cross-species comparisons, 18 s rRNA and β-actin were the most stable (0.05 stability). Differences in HSP up-regulation across temperature treatments were determined with a one-way Analysis of Variance (ANOVA) in which fold expression values were log_10_ transformed to meet assumptions of normality. Significant up-regulation relative to controls was identified with *post hoc* Tukey tests.

### Availability of supporting data

The data sets supporting the results of this article are included in the Dryad Digital repository (https://datadryad.org/resource/doi:10.5061/dryad.8vj6j) [66], within the article and its additional files.

## Additional files

Additional file 1: Figure S1. Maximum likelihood phylogeny of *Hsp60* (mitochondrial form) for 17 species of insects (rooted on *A. pisum*) using a JTT amino acid substitution model and 1000 bootstraps replicates. (DOCX 233 kb) Additional file 2: Figure S2. Maximum likelihood phylogeny of *hsc70-3* (BIP) for 17 species of insects (rooted on *A. pisum*) using a JTT amino acid substitution model and 1000 bootstrap replicates. (DOCX 230 kb) Additional file 3: Figure S3. Maximum likelihood phylogeny of *hsc70-5* for 17 species of insects (rooted on *A. pisum*) using a JTT amino acid substitution model and 1000 bootstrap replicates. (DOCX 240 kb) Additional file 4: Figure S4. Local alignment of the genomic region of orthologous *hsp83* from 17 insect species spanning 5 insect Orders. (DOCX 89 kb) Additional file 5: Figure S5. Local alignment of the genomic region of orthologous *hsc70-4* from 17 insect species spanning 5 insect Orders. (DOCX 231 kb) Additional file 6: Figure S6. Local alignment of the genomic region of orthologous *hsp40* from 17 insect species spanning 5 insect Orders. (DOCX 97 kb) Additional file 7: Figure S7. Percent survival (+/1 SD) of *Aphaenogaster picea* and *P. barbatus* (right panel) from heat shock treatments at different temperature treatments. (DOCX 79 kb) Additional file 8: Table S1. Nucleotide sequences used to characterize the molecular evolution of heat shock proteins. (DOCX 138 kb) Additional file 9: Table S2. Sequence annotations (position, length, arrangement) of cis-regulatory HSEs for each HSP gene and across all species screened. (DOCX 177 kb) Additional file 10: Table S3. Primer sets for qPCR including housekeeping and heat shock genes. (DOCX 79 kb)

## Abbreviations

ANOVA: Analysis of Variance
Hsp: heat shock protein
HSR: heat shock response
HSE: heat shock element
HSF: heat shock factor
qPCR: quantitative polymerase chain reaction
