# Supplementary material for: The evolution of heat shock protein sequences, cis-regulatory elements, and expression profiles in the eusocial Hymenoptera
Source: BMC Evol Biol. 2016 Jan 19;16:15. doi: 10.1186/s12862-015-0573-0 (PMC4717527; doi:10.1186/s12862-015-0573-0)
Supplement: Additional file 1: Figure S1. — Maximum likelihood phylogeny of Hsp60 (mitochondrial form) for 17 species of insects (rooted on A. pisum) using a JTT amino acid substitution model and 1000 bootstraps replicates. (DOCX 233 kb) [file 12862_2015_573_MOESM1_ESM.docx]

Figure S1. Maximum likelihood phylogeny of HSP60 (mitochondrial form) for 17 species of insects (rooted on *A. pisum*) using a JTT amino acid substitution model and 1000 bootstraps. Locally aligned cis-regulatory HSEs 700 base pairs upstream the predicted TSS are mapped back to the phylogeny and display little conservation in position.
