# Supplementary material for: The evolution of heat shock protein sequences, cis-regulatory elements, and expression profiles in the eusocial Hymenoptera
Source: BMC Evol Biol. 2016 Jan 19;16:15. doi: 10.1186/s12862-015-0573-0 (PMC4717527; doi:10.1186/s12862-015-0573-0)
Supplement: Additional file 2: Figure S2. — Maximum likelihood phylogeny of hsc70-3 (BIP) for 17 species of insects (rooted on A. pisum) using a JTT amino acid substitution model and 1000 bootstrap replicates. (DOCX 230 kb) [file 12862_2015_573_MOESM2_ESM.docx]

Figure S2. Maximum likelihood phylogeny of *hsc70-3* (BIP) for 17 species of insects (rooted on *A. pisum*) using a JTT amino acid substitution model and 1000 bootstraps. Locally aligned cis-regulatory HSEs 500 base pairs upstream the predicted TSS were mapped back to the phylogeny and display little conservation in position.
