# Supplementary material for: The evolution of heat shock protein sequences, cis-regulatory elements, and expression profiles in the eusocial Hymenoptera
Source: BMC Evol Biol. 2016 Jan 19;16:15. doi: 10.1186/s12862-015-0573-0 (PMC4717527; doi:10.1186/s12862-015-0573-0)
Supplement: Additional file 4: Figure S4. — Local alignment of the genomic region of orthologous hsp83 from 17 insect species spanning 5 insect Orders. (DOCX 89 kb) [file 12862_2015_573_MOESM4_ESM.docx]

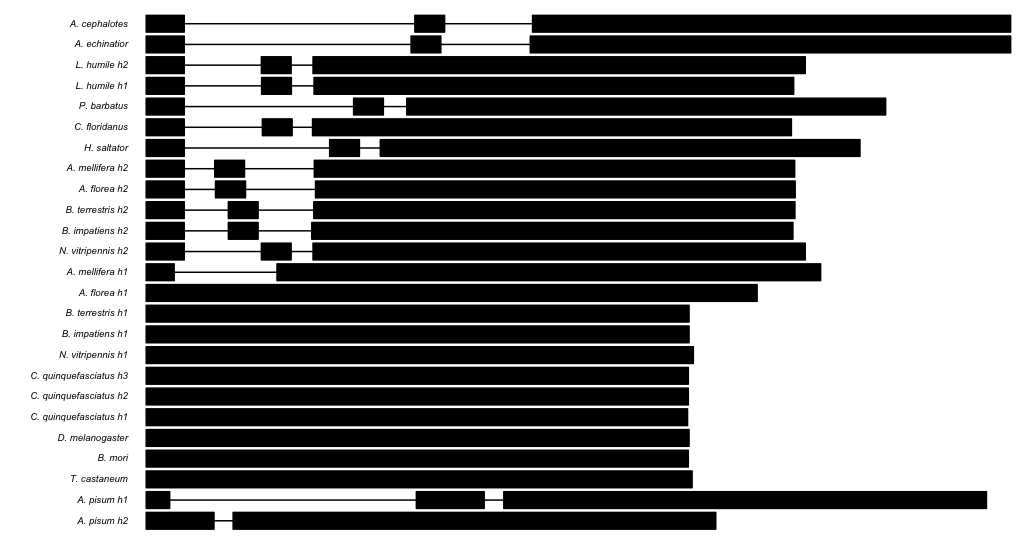


Figure S4. Local alignment of the genomic region of orthologous *hsp83* from 17 insect species spanning 5 orders. Exons and introns are represented as boxes and lines, respectively.
