# Supplementary material for: The evolution of heat shock protein sequences, cis-regulatory elements, and expression profiles in the eusocial Hymenoptera
Source: BMC Evol Biol. 2016 Jan 19;16:15. doi: 10.1186/s12862-015-0573-0 (PMC4717527; doi:10.1186/s12862-015-0573-0)
Supplement: Additional file 7: Figure S7. — Percent survival (+/1 SD) of Aphaenogaster picea and P. barbatus (right panel) from heat shock treatments at different temperature treatments. (DOCX 79 kb) [file 12862_2015_573_MOESM7_ESM.docx]

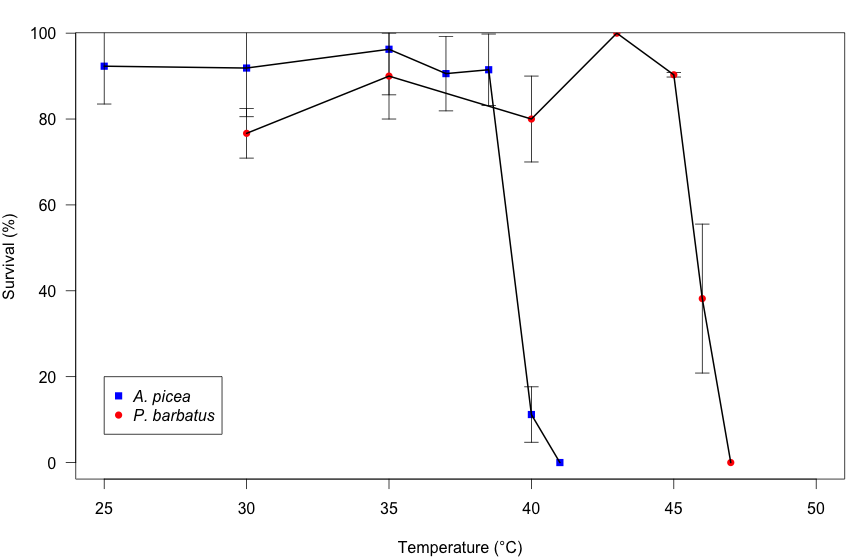


Figure S7. Percent survival (+/1 SD) of *Aphaenogaster picea* and *P. barbatus* (right panel) from heat shock treatments at different temperature treatments. Thermal tolerance experiments included 10 individuals per treatment, replicated across 8 colonies for *A. picea* and 3 colonies for *P. barbatus*.
