# Supplementary material for: The evolution of heat shock protein sequences, cis-regulatory elements, and expression profiles in the eusocial Hymenoptera
Source: BMC Evol Biol. 2016 Jan 19;16:15. doi: 10.1186/s12862-015-0573-0 (PMC4717527; doi:10.1186/s12862-015-0573-0)
Supplement: Additional file 8: Table S1. — Nucleotide sequences used to characterize the molecular evolution of heat shock proteins. (DOCX 138 kb) [file 12862_2015_573_MOESM8_ESM.docx]

Table S1. Nucleotide sequences used to characterize the molecular evolution of heat shock proteins.

| **Gene** | **Species** | **Sequence Source** | **Nucleotide Sequence ID/ Accension#** | **Genomic Region(Scaffold ID)** |
| --- | --- | --- | --- | --- |
| hsc70_h4-1 | *A. cephalotes* | Fourmidable | lcl\|ACEP_00004092-RA | scaffold00008_ |
| hsc70_h4-1 | *A. echinatior* | Fourmidable | lcl\|Aech_00116 | lcl\|Aech_gn2.0_scaffold121 |
| hsc70_h4-1 | *C. floridanus* | Fourmidable | lcl\|Cflo_09392--XP_392933.2_APIME | lcl\|Cflo_gn3.3_scaffold299 |
| hsc70_h4-1 | *H. saltator* | Fourmidable | lcl\|Hsal_05193--XP_392933.2_APIME | scaffold287 |
| hsc70_h4-1 | *L humile* | Fourmidable | lcl\|LH12354-RA | scf7180001004585 |
| hsc70_h4-1 | *P. barbatus* | Fourmidable | lcl\|pbar_HSP70-1 | lcl\|pbar_scf7180000350168 |
| hsc70_h4-1 | *S. invicta* | Fourmidable | lcl\|SI2.2.0_15088 | Si_gnF.scaffold06738 |
| hsc70_h4-1 | *A. florea* | Genbank | XM_003690610.1 | GL575310.1 |
| hsc70_h4-1 | *A. mellifera* | Genbank | NM_001160050.1 | AADG06000220.1 |
| hsc70_h4-1 | *B. impatiens* | Genbank | XM_003485778.1 | AEQM02001076.1 |
| hsc70_h4-1 | *B. terrestris* | Genbank | XM_003397414.1 | GL898810.1 |
| hsc70_h4-1 | *N. vitripennis* | Genbank | NM_001172757.1 | AAZX01007508.1 |
| hsc70_h4-2 | *A. cephalotes* | Fourmidable | lcl\|ACEP_00003321-RA | scaffold00006 |
| hsc70_h4-2 | *A. echinatior* | Fourmidable | lcl\|Aech_07432 | scaffold447 |
| hsc70_h4-2 | *C. floridanus* | Fourmidable | lcl\|Cflo_07424--XP_623130.1_APIME | lcl\|Cflo_gn3.3_scaffold992 |
| hsc70_h4-2 | *H. saltator* | Fourmidable | lcl\|ACEP_00003321-RA | scaffold304 |
| hsc70_h4-2 | *L humile* | Fourmidable | lcl\|LH21302-RA | scf7180001005005 |
| hsc70_h4-2 | *P. barbatus* | Fourmidable | lcl\|PB19955-RA | lcl\|pbar_scf7180000350291 |
| hsc70_h4-2 | *S. invicta* | Fourmidable | lcl\|SI2.2.0_04536 | lcl\|Si_gnF.scaffold10523 |
| hsc70_h4-2 | *A. florea* | Genbank | XM_003696016.1 | GL577044.1 |
| hsc70_h4-2 | *A. mellifera* | Genbank | NM_001160072.1 | AADG06003252.1 |
| hsc70_h4-2 | *B. impatiens* | Genbank | XM_003487055.1 | AEQM02002359.1 |
| hsc70_h4-2 | *B. terrestris* | Genbank | XM_003396727.1 | GL898803.1 |
| hsc70_h4-2 | *N. vitripennis* | Genbank | XM_001607994.2 | NW_001815126.1 |
| hsc70-4 | *T. castaneum* | Genbank | XM_961518.2 | AAJJ01001581.1 |
| hsc70-4 | *D. melanogaster* | Genbank | NM_079632.5 | 3R:11060370,11072336 |
| hsc70-4_h1 | *C. quinquefasciatus* | Genbank | XM_001848166.1 | AAWU01010038.1 |
| hsc70-4_h2 | *C. quinquefasciatus* | Genbank | XM_001850475.1 | AAWU01014529.1 |
| hsc70-4 | *B. mori* | Genbank | HQ694956.1 | AADK01001426.1 |
| hsc70-4_h1 | *A. pisum* | Genbank | XM_001951172.2 | ABLF02013051.1 |
| hsc70-4_h2 | *A. pisum* | Genbank | XM_001951351.2 | ABLF02026083.1 |
| hsp83 | *A. cephalotes* | Fourmidable | lcl\|ACEP_00012985-RA | lcl\|scaffold00044 |
| hsp83 | *A. echinatior* | Fourmidable | lcl\|Aech_04253 | lcl\|Aech_gn2.0_scaffold482 |
| hsp83 | *C. floridanus* | Fourmidable | lcl\|Cflo_03666--XP_623939.1_APIME | lcl\|Cflo_gn3.3_scaffold714 |
| hsp83 | *H. saltator* | Fourmidable | lcl\|Hsal_04205--XP_623939.1_APIME | lcl\|Hsal_gn3.3_scaffold129 |
| hsp83_1 | *L humile* | Fourmidable | lcl\|LH15144-RA | lcl\|scf7180001004854 |
| hsp83_2 | *L humile* | Fourmidable | lcl\|LH22981-RA | scf7180001005039 |
| hsp83 | *P. barbatus* | Fourmidable | lcl\|pbar_hsp90-1 | lcl\|pbar_scf7180000350230 |
| hsp83_h1 | *A. florea* | Genbank | XR_142802.1 | GL576082.1 |
| hsp83_h2 | *A. florea* | Genbank | XM_003694884.1 | GL576665.1 |
| hsp83_h1 | *A. mellifera* | Genbank | NM_001160064.1 | NM_001160064.1 |
| hsp83_h2 | *A. mellifera* | Genbank | XM_395168.4 | GL630340.1 |
| hsp83_h1 | *B. impatiens* | Genbank | XM_003486590.1 | GL739246.1 |
| hsp83_h2 | *B. impatiens* | Genbank | XM_003492101.1 | GL739317.1 |
| hsp83_h1 | *B. terrestris* | Genbank | XM_003396849.1 | AELG01004018.1 |
| hsp83_h2 | *B. terrestris* | Genbank | XM_003393081.1 | GL898771.1 |
| hsp83_h1 | *N. vitripennis* | Genbank | XM_001601080.2 | AAZX01021497.1 |
| hsp83_h2 | *N. vitripennis* | Genbank | XM_001605141.2 | AAZX01012164.1 |
| hsp83_h1 | *A. pisum* | Genbank | XM_001944726.2 | ABLF02011313.1 |
| hsp83_h2 | *A. pisum* | Genbank | XM_001943137.2 | ABLF02034733.1 |
| hsp83 | *B. mori* | Genbank | NM_001043411.1 | AADK01011786.1 |
| hsp83_h1 | *C. quinquefasciatus* | Genbank | XM_001865449.1 | AAWU01027867.1 |
| hsp83_h2 | *C. quinquefasciatus* | Genbank | XM_001861893.1 | AAWU01019716.1 |
| hsp83_h3 | *C. quinquefasciatus* | Genbank | XM_001861891.1 | AAWU01019715.1 |
| hsp83 | *D. melanogaster* | Genbank | NM_079175.3 | 3L:3185969,3198969 |
| hsp83 | *T. castaneum* | Genbank | EF633444.1 | AAJJ01000764.1 |
| hsc70-3(BIP) | *A. cephalotes* | Fourmidable | lcl\|ACEP_00004082-RA | scaffold00008 |
| hsc70-3(BIP) | *A. echinatior* | Fourmidable | lcl\|Aech_00126 | lcl\|Aech_gn2.0_scaffold121 |
| hsc70-3(BIP) | *C. floridanus* | Fourmidable | lcl\|Cflo_02717--XP_393090.3_APIME | lcl\|Cflo_gn3.3_scaffold2159 |
| hsc70-3(BIP) | *H. saltator* | Fourmidable | lcl\|Hsal_01928--XP_393090.3_APIME | lcl\|Hsalt_gn3.3_scaffold12 |
| hsc70-3(BIP) | *L humile* | Fourmidable | lcl\|LH23404-RA | lcl\|scf7180001005056 |
| hsc70-3(BIP) | *P. barbatus* | Fourmidable | lcl\|PB15578-RA | lcl\|pbar_scf7180000350168 |
| hsc70-3(BIP) | *S. invicta* | Fourmidable | lcl\|SI2.2.0_10004 | lcl\|Si_gnF.scaffold06738 |
| hsc70-3(BIP) | *A. florea* | Genbank | XM_003698615.1 | AEKZ01013723.1 |
| hsc70-3(BIP) | *A. mellifera* | Genbank | NM_001160052.1 | AADG06004363.1 |
| hsc70-3(BIP) | *B. impatiens* | Genbank | XM_003490850.1 | NT_176897.1 |
| hsc70-3(BIP) | *B. terrestris* | Genbank | XM_003394262.1 | NC_015764.1 |
| hsc70-3(BIP) | *N. vitripennis* | Genbank | XM_001606413.3 | NW_001820638.3 |
| hsc70-3(BIP) | *A. pisum* | Genbank | NM_001162948.1 | ABLF02039326.1 |
| hsc70-3(BIP) | *B. mori* | Genbank | gb\|JF836796.1 | AADK01000639.1 |
| hsc70-3(BIP) | *C. quinquefasciatus* | Genbank | XM_001845166.1 | AAWU01004826.1 |
| hsc70-3(BIP) | *D. melanogaster* | Genbank | NP_727563.1 | NC_004354.4 |
| hsc70-3(BIP) | *T. castaneum* | Genbank | XM_965476.2 | AAJJ01001199.1 |
| hsc70-5 | *A. cephalotes* | Fourmidable | lcl\|ACEP_00000449-RA | lcl\|scaffold00001 |
| hsc70-5 | *A. echinatior* | Fourmidable | lcl\|Aech_03006 | lcl\|Aech_gn2.0_scaffold159 |
| hsc70-5 | *C. floridanus* | Fourmidable | lcl\|Cflo_00087--XP_392147.2_APIME | lcl\|Cflo_gn3.3_scaffold107 |
| hsc70-5 | *H. saltator* | Fourmidable | lcl\|Hsal_04386--XP_392147.2_APIME | lcl\|Hsal_gn3.3_scaffold61 |
| hsc70-5 | *L humile* | Fourmidable | lcl\|LH22708-RA | lcl\|scf7180001005031 |
| hsc70-5 | *P. barbatus* | Fourmidable | lcl\|PB16617-RA | lcl\|scf718000350202 |
| hsc70-5 | *S. invicta* | Fourmidable | lcl\|SI2.2.0_04074 | lcl\|Si_gnF.scaffold06131 |
| hsc70-5 | *A. florea* | Genbank | XM_003695561.1 | NW_003791058.1 |
| hsc70-5 | *A. mellifera* | Genbank | NM_001160048.1 | NW_003378185.1 |
| hsc70-5 | *B. impatiens* | Genbank | XM_003492464.1 | NT_177158.1 |
| hsc70-5 | *B. terrestris* | Genbank | XM_003393301.1 | NW_003565321.1 |
| hsc70-5 | *N. vitripennis* | Genbank | XM_001599475.3 | NW_001816026.1 |
| hsc70-5 | *A. pisum* | Genbank | XM_001950464.3 | NW_003383549.1 |
| hsc70-5 | *B. mori* | Genbank | AK386403.1 | NW_004581849.1 |
| hsc70-5 | *C. quinquefasciatus* | Genbank | XM_001845988.1 | NW_001886767.1 |
| hsc70-5 | *D. melanogaster* | Genbank | NM_079017.2 | NT_033778.4 |
| hsc70-5 | *T. castaneum* | Genbank | XM_970293.2 | NW_001092777.1 |
| hsp60 | *A. cephalotes* | Fourmidable | lcl\|ACEP_00009250-RA | lcl\|scaffold00024 |
| hsp60 | *A. echinatior* | Fourmidable | lcl\|Aech_15304 | lcl\|Aech_gn2.0_scaffold577 |
| hsp60 | *C. floridanus* | Fourmidable | lcl\|Cflo_06234--XP_392899.2_APIME | cflo_scaffold1177 |
| hsp60 | *H. saltator* | Fourmidable | lcl\|Hsal_06496--XP_392899.2_APIME | lcl\|Hsal_gn3.3_scaffold846 |
| hsp60 | *L humile* | Fourmidable | lcl\|LH18427-RA | L_humile_lcl\|scf7180001004948 |
| hsp60 | *P. barbatus* | Fourmidable | lcl\|PB24919-RA | lcl\|pbar_scf7180000350375 |
| hsp60 | *S. invicta* | Fourmidable | lcl\|SI2.2.0_14312 | Si_gnF.scaffold06131 |
| hsp60 | *A. florea* | Genbank | XM_003691238.1 | AEKZ01002697.1 |
| hsp60 | *A. mellifera* | Genbank | XM_392899.4 | AADG06003748.1 |
| hsp60 | *B. impatiens* | Genbank | XM_003491816.1 | NT_177059.1 |
| hsp60 | *B. terrestris* | Genbank | XM_003399581.1 | NC_015773.1 |
| hsp60 | *N. vitripennis* | Genbank | XM_003427885.1 | NW_001820471.1 |
| hsp60 | *A. pisum* | Genbank | XM_003241657.1 | NW_003383549.1 |
| hsp60 | *B. mori* | Genbank | XM_004923900.1 | NW_004581704.1 |
| hsp60 | *C. quinquefasciatus* | Genbank | XM_001850449.1 | NW_001886908.1 |
| hsp60 | *D. melanogaster* | Genbank | NM_078560.3 | NC_004354.4 |
| hsp60 | *T. castaneum* | Genbank | XM_966537.2 | NW_001092828.1 |
| hsp40 | *A. cephalotes* | Fourmidable | lcl\|ACEP_00015479-RA | lcl\|scaffold00081 |
| hsp40 | *A. echinatior* | Fourmidable | lcl\|Aech_03889 | lcl\|Aech_gn2.0_scaffold220 |
| hsp40 | *C. floridanus* | Fourmidable | lcl\|Cflo_11071--XP_394545.2_APIME | lcl\|Cflo_gn3.3_scaffold2366 |
| hsp40 | *H. saltator* | Fourmidable | lcl\|Hsal_00950--XP_394545.2_APIME | lcl\|Hsal_gn3.3_scaffold347 |
| hsp40 | *L humile* | Fourmidable | lcl\|LH19907-RA | L humile lcl\|scf7180001004978 |
| hsp40 | *P. barbatus* | Fourmidable | lcl\|PB12157-RA | lcl\|pbar_scf7180000349945 |
| hsp40 | *S. invicta* | Fourmidable | lcl\|SI2.2.0_05719 | Si_gnF.scaffold05549 |
| hsp40 | *A. florea* | Genbank | XM_003696250.1 | AEKZ01010575.1 |
| hsp40 | *A. mellifera* | Genbank | XM_394545.4 | NC_007074.3 |
| hsp40 | *B. impatiens* | Genbank | XM_003492696.1 | AEQM02008753.1 |
| hsp40 | *B. terrestris* | Genbank | XM_003395466.1 | AELG01000977.1 |
| hsp40 | *N. vitripennis* | Genbank | XM_003427542.1 | NW_001819015.1 |
| hsp40 | *A. pisum* | Genbank | XM_001949026.2 | XM_001949026.2 |
| hsp40 | *B. mori* | Genbank | FJ592078.1 | AADK01009415.1 |
| hsp40 | *C. quinquefasciatus* | Genbank | XM_001845411.1 | NW_001886754.1 |
| hsp40 | *D. melanogaster* | Genbank | NM_001258903.1 | NM_001258903.1 |
| hsp40 | *T. castaneum* | Genbank | XM_971038.2 | XM_971038.2 |
