# Supplementary material for: The evolution of heat shock protein sequences, cis-regulatory elements, and expression profiles in the eusocial Hymenoptera
Source: BMC Evol Biol. 2016 Jan 19;16:15. doi: 10.1186/s12862-015-0573-0 (PMC4717527; doi:10.1186/s12862-015-0573-0)
Supplement: Additional file 9: Table S2. — Sequence annotations (position, length, arrangement) of cis-regulatory HSEs for each HSP gene and across all species screened. (DOCX 177 kb) [file 12862_2015_573_MOESM9_ESM.docx]

Table S2. Sequence annotations of cis-regulatory HSEs for each HSP gene and across all species screened. The 5’ and 3’ positions correspond to the number of basepairs away from the transcription start site.

| n | Gene | Species | 5' | 3' | Length_Type | Sequence |
| --- | --- | --- | --- | --- | --- | --- |
| 1 | hsp83 | *Acromyrmex echinatior* | -65 | -45 | 4G | GGCGCCTTCTAGAATATTCG |
| 2 | hsp83 | *Acromyrmex echinatior* | -100 | -85 | 3G | AGAAGGTTCTAGAAG |
| 3 | hsp83 | *Acromyrmex echinatior* | -325 | -300 | 5G | GGAGCTATCGCGATGGTTCTAGATC |
| 4 | hsp83 | *Apis florea h1* | -56 | -36 | 4G | GGTGCCTTCTAGAATTTTCA |
| 5 | hsp83 | *Apis florea h1* | -65 | -50 | 3T | CTTCCGGCAGGTGCC |
| 6 | hsp83 | *Apis florea h1* | -89 | -74 | 3G | AGAATGTTCTAGAAA |
| 7 | hsp83 | *Apis florea h1* | -315 | -290 | 5G | GGAGGTATCGCGATGTTTCTAGACA |
| 8 | hsp83 | *Apis florea h2* | -71 | -31 | 8T | GTTCTAGAACATTCTAGAACATTCTAGAATTTTAGTGAGT |
| 9 | hsp83 | *Apis florea h2* | -289 | -274 | 3T | ATGCCGGAAGTATCA |
| 10 | hsp83 | *Apis mellifera H1* | -56 | -36 | 4G | GGTGCCTTCTAGAATTTTCA |
| 11 | hsp83 | *Apis mellifera H1* | -65 | -50 | 3T | CTTCCGGCAGGTGCC |
| 12 | hsp83 | *Apis mellifera H1* | -89 | -74 | 3G | AGAATGTTCTAGAAA |
| 13 | hsp83 | *Apis mellifera H1* | -314 | -289 | 5G | GGAGGTATCGCGATGTTTCTAGACA |
| 14 | hsp83 | *Apis mellifera h2* | -71 | -31 | 8T | GTTCTAGAACATTCTAGAACATTCTAGAATTTTAGTGAGT |
| 15 | hsp83 | *Apis mellifera h2* | -285 | -270 | 3T | ATACCGGAAGTATCA |
| 16 | hsp83 | *Atta cephalotes* | -65 | -40 | 5G | GGCGCCTTCTAGAATATTCGTGTTC |
| 17 | hsp83 | *Atta cephalotes* | -100 | -85 | 3G | AGAAGGTTCTAGAAG |
| 18 | hsp83 | *Atta cephalotes* | -327 | -302 | 5G | GGAGCTATCGCGATGGTTCTAGATC |
| 19 | hsp83 | *Bombus impatiens* | -56 | -36 | 4G | GGTGCCTTCTAGAATTTTCA |
| 20 | hsp83 | *Bombus impatiens* | -65 | -50 | 3T | CTTCCGGCAGGTGCC |
| 21 | hsp83 | *Bombus impatiens* | -89 | -74 | 3G | AGAATGTTCTAGAAA |
| 22 | hsp83 | *Bombus impatiens* | -313 | -288 | 5G | CGAGGTATCGCGATGTTTCTAGACA |
| 23 | hsp83 | *Bombus impatiens h2* | -69 | -39 | 6T | GTTCTAGAACATTCTAGAACATTCTAGAAT |
| 24 | hsp83 | *Bombus impatiens h2* | -283 | -268 | 3T | ATTCCGGAAGTACCG |
| 25 | hsp83 | *Bombus terrestris* | -56 | -36 | 4G | GGTGCCTTCTAGAATTTTCA |
| 26 | hsp83 | *Bombus terrestris* | -65 | -50 | 3T | CTTCCGGCAGGTGCC |
| 27 | hsp83 | *Bombus terrestris* | -89 | -74 | 3G | AGAATGTTCTAGAAA |
| 28 | hsp83 | *Bombus terrestris* | -313 | -288 | 5G | CGAGGTATCGCGATGTTTCTAGACA |
| 29 | hsp83 | *Bombus terrestris h2* | -71 | -41 | 6T | GTTCTAGAACATTCTAGAACATTCTAGAAT |
| 30 | hsp83 | *Bombus terrestris h2* | -285 | -270 | 3T | ATTCCGGAAATACCG |
| 31 | hsp83 | *Bombyx mori* | -106 | -76 | 6T | TTTCTAGAATATTCAGGAATGTTCCAGATA |
| 32 | hsp83 | *Camponotus floridanus* | -61 | -31 | 6T | GTTCTCAAACCTTCTAGAATCTTCAGCACT |
| 33 | hsp83 | *Camponotus floridanus* | -90 | -75 | 3G | AGAAGCTTCTAGAAG |
| 34 | hsp83 | *Camponotus floridanus* | -313 | -288 | 5G | CGAGCTATCGCGATGGTTCTAGATC |
| 35 | hsp83 | *Drosophila melanogaster* | -84 | -44 | 8T | CATCCAGAAGCCTCTAGAAGTTTCTAGAGACTTCCAGTTC |
| 36 | hsp83 | *Harpegnathos saltator* | -63 | -43 | 4T | GTTCGAGAAGCTTCTAGAAC |
| 37 | hsp83 | *Harpegnathos saltator* | -92 | -77 | 3G | AGAATGTTCTAGAAG |
| 38 | hsp83 | *Harpegnathos saltator* | -309 | -289 | 4T | CATCGCGATGATTCTAGATT |
| 39 | hsp83 | *Linepithema humile h1* | -91 | -71 | 4T | TTGCTCAAAGATTCGTGCGC |
| 40 | hsp83 | *Linepithema humile h1* | -194 | -169 | 5G | CGAACTATCGCGATGGTTCTAGACT |
| 41 | hsp83 | *Linepithema humile h1* | -207 | -192 | 3T | GTTCCCGCTTGTTCG |
| 42 | hsp83 | *Linepithema humile h1* | -331 | -311 | 4G | GGAATGTTTATGACGCTCCT |
| 43 | hsp83 | *Linepithema humile H2* | -64 | -34 | 6G | CGAATCTTCTAGAATATTGTAGAATATTCA |
| 44 | hsp83 | *Linepithema humile H2* | -98 | -83 | 3G | AGAACATTCTAGAAG |
| 45 | hsp83 | *Linepithema humile H2* | -320 | -295 | 5G | CGAGCTATCGCGATAGTTCTAGACG |
| 46 | hsp83 | *Nasonia vitripennis h1* | -56 | -41 | 3G | AGAATTTTCTAGACT |
| 47 | hsp83 | *Nasonia vitripennis h1* | -89 | -74 | 3G | GGAAAGTTCGAGAAG |
| 48 | hsp83 | *Nasonia vitripennis h1* | -192 | -177 | 3G | CGAAACCTCCTGAAA |
| 49 | hsp83 | *Nasonia vitripennis h1* | -300 | -280 | 4T | CCCCGCGAAGCTTCCGGCGC |
| 50 | hsp83 | *Nasonia vitripennis h1* | -330 | -310 | 4T | GCTCGAGACGTTTCTAGACC |
| 51 | hsp83 | *Nasonia vitripennis h2* | -87 | -47 | 8T | GTTCTGGAAGCTTCTAGAAGGTTCTGGAAGGTTCGAGACG |
| 52 | hsp83 | *Pogonomyrmex barbatus h1* | -51 | -36 | 3T | TTTCTAGAATATTCG |
| 53 | hsp83 | *Pogonomyrmex barbatus h1* | -80 | -65 | 3G | AGAACATTCTAGAAG |
| 54 | hsp83 | *Pogonomyrmex barbatus h1* | -303 | -273 | 6G | CGAGTAATCGCGATGGTTCTAGATTCTCCT |
| 55 | hsp83 | *Tribolium castaneum* | -86 | -41 | 9G | CGAAGCGTCGGAAAGCGTCTAGATTCTTCTCGAATAATCTAGAAC |
| 56 | hsp83 | *Tribolium castaneum* | -186 | -166 | 4G | GGATTTTTCTAGAAAAATCA |
| 57 | BIP | *Acromyrmex echinatior* | -370 | -350 | 4G | CGGAGCGTCTGGAATTATCA |
| 58 | BIP | *Acromyrmex echinatior* | -475 | -450 | 5G | CGAGTCTTATAGAAAACTCCCGCGT |
| 59 | BIP | *Atta cephalotes* | -489 | -469 | 4G | TGGAGCGTCTGGAATTATCT |
| 60 | BIP | *Bombyx mori* | -162 | -147 | 3G | AGAATTTTCTCGACG |
| 61 | BIP | *Camponotus floridanus* | -145 | -130 | 3T | GTTCGCGAGTAATCT |
| 62 | BIP | *Camponotus floridanus* | -424 | -409 | 3T | ATACGAGAACGTTCC |
| 63 | BIP | *Camponotus floridanus* | -191 | -171 | 4G | CGAAGCTCGTGGAAATTTCC |
| 64 | BIP | *Camponotus floridanus* | -410 | -390 | 4G | CGTCGAGAATCTTCCTGCCA |
| 65 | BIP | *Camponotus floridanus* | -511 | -491 | 4T | CCTCGGGAAGAGTCGAGAGG |
| 66 | BIP | *Culex quinquefasciatus* | -489 | -469 | 4G | TGCCAACCACTTTCTCGAAC |
| 67 | BIP | *Culex quinquefasciatus* | -89 | -64 | 5T | TTTCGGGAACATTCCAGAGTATCCC |
| 68 | BIP | *Drosophila melanogaster* | -89 | -74 | 3G | CGATGTTTCTAGAAA |
| 69 | BIP | *Harpegnathos saltator* | -68 | -53 | 3G | GGAATCTTCCCGGAA |
| 70 | BIP | *Harpegnathos saltator* | -359 | -344 | 3T | GGAATATTCCAGTCG |
| 71 | BIP | *Harpegnathos saltator* | -532 | -511 | 4T | TTTCCCGATCTCTCTCGCTCA |
| 72 | BIP | *Linepithema humile* | -200 | -185 | 3G | CGAACACGCGCGAAT |
| 73 | BIP | *Nasonia vitripennis* | -191 | -166 | 5T | CGACCCGAAGCTTCTAGAAGCGGCG |
| 74 | BIP | *Pogonomyrmex barbatus* | -24 | -9 | 3T | ATTCGCGGACCGTCC |
| 75 | BIP | *Pogonomyrmex barbatus* | -191 | -176 | 3T | TTTCTAGAATCATCT |
| 76 | BIP | *Pogonomyrmex barbatus* | -286 | -271 | 3T | TCTCGCGGAAATTCC |
| 77 | BIP | *Solenopsis invicta* | -519 | -489 | 6T | GCGCGTCGAACTTCTGGAATCATCTCGTCG |
| 78 | BIP | *Tribolium castaneum* | -222 | -207 | 3G | TGACTCTTCTGGTAT |
| 79 | BIP | *Tribolium castaneum* | -85 | -70 | 3T | GATCCAGAACTTTCA |
| 80 | BIP | *Tribolium castaneum* | -191 | -176 | 3T | TGGCTTGAACATTCC |
| 81 | BIP | *Tribolium castaneum* | -161 | -136 | 5T | GTCCCGGAACATTCAGGAAACTTCA |
| 82 | BIP | *Tribolium castaneum* | -416 | -391 | 5T | ATGCAAAAATTTTCAAGTTGATGCG |
| 83 | hsc70-4 | *Acromyrmex echinatior h1* | -62 | -37 | 5G | GGAACATTCCAGAAGCTTGCCGCAT |
| 84 | hsc70-4 | *Acromyrmex echinatior h1* | -77 | -62 | 3G | TGAGTCTGCGCGGAA |
| 85 | hsc70-4 | *Acromyrmex echinatior h1* | -152 | -107 | 9G | CGATGCCTCCCGACCAATCGCGCGCCTACGCAAACGTGCGTGAAC |
| 86 | hsc70-4 | *Acromyrmex echinatior h1* | -301 | -276 | 5T | ACTCGGGAAAATTCGCGTATACTCG |
| 87 | hsc70-4 | *Acromyrmex echinatior h1* | -516 | -486 | 6G | AGTAGTTTCTAGAATTTGTTCGATGCGTCC |
| 88 | hsc70-4 | *Acromyrmex echinatior h2* | -62 | -47 | 3G | AGAAGCTTCTCGAAG |
| 89 | hsc70-4 | *Acromyrmex echinatior h2* | -112 | -67 | 9G | GGAAGCTTCTCGAATGATCTCGAGGACTCGCGCCGTGATTGGTCA |
| 90 | hsc70-4 | *Acromyrmex echinatior h2* | -213 | -198 | 3T | AGCCGTGAATCTTCT |
| 91 | hsc70-4 | *Acyrthosiphon pisum h1* | -202 | -167 | 7T | TTCCATGAAACGTCTAGAATGTTCTAGAGCCGTCG |
| 92 | hsc70-4 | *Apis florea h1* | -63 | -43 | 4G | AGAACATTCCAGAAGCGTCC |
| 93 | hsc70-4 | *Apis florea h1* | -82 | -67 | 3G | AGAATGTGCTAGAGT |
| 94 | hsc70-4 | *Apis florea h1* | -495 | -480 | 3G | AGATGTTACAGGAAT |
| 95 | hsc70-4 | *Apis florea h2* | -59 | -44 | 3G | AGAAACTTCTCGAAG |
| 96 | hsc70-4 | *Apis florea h2* | -84 | -64 | 4G | CGAAATGCCGTGATTGGTCA |
| 97 | hsc70-4 | *Apis florea h2* | -136 | -121 | 3T | GTTCTAGATGCATCG |
| 98 | hsc70-4 | *Apis florea h2* | -222 | -202 | 4T | ATTCCAGCATTTTCTCGTAT |
| 99 | hsc70-4 | *Apis florea h2* | -280 | -265 | 3G | TGAATAAACAAGAAA |
| 100 | hsc70-4 | *Apis melifera h2* | -59 | -44 | 3G | AGAAACTTCTCGAAG |
| 101 | hsc70-4 | *Apis melifera h2* | -84 | -64 | 4G | TGAAATGCCGTGATTGGTCA |
| 102 | hsc70-4 | *Apis melifera h2* | -136 | -121 | 3T | GTTCTAGATGCATCG |
| 103 | hsc70-4 | *Apis melifera h2* | -222 | -202 | 4T | ATTCCAGCATTTTCTCGTAT |
| 104 | hsc70-4 | *Apis mellifera h1* | -63 | -43 | 4G | AGAACATTCCAGAAGCGTCC |
| 105 | hsc70-4 | *Apis mellifera h1* | -82 | -67 | 3G | AGAATGTGCTAGAGT |
| 106 | hsc70-4 | *Apis mellifera h1* | -486 | -471 | 3G | AGATATTACAAGAAT |
| 107 | hsc70-4 | *Atta cephalotes h1* | -62 | -37 | 5G | GGAACATTCCAGAAGCTTGCCGCAT |
| 108 | hsc70-4 | *Atta cephalotes h1* | -77 | -62 | 3G | TGAGTCTGCGCGGAA |
| 109 | hsc70-4 | *Atta cephalotes h1* | -152 | -107 | 9G | CGATGCCTCCCGACCAATCGCGCGCCTACGCAAACGTGCGTGAAC |
| 110 | hsc70-4 | *Atta cephalotes h1* | -301 | -276 | 5T | ATTCGAGAAGATTCGCGTATACTCG |
| 111 | hsc70-4 | *Atta cephalotes h1* | -527 | -512 | 3G | AGTAGTTTCTAGAAT |
| 112 | hsc70-4 | *Atta cephalotes h2* | -62 | -47 | 3G | AGAAGCTTCTCGAAG |
| 113 | hsc70-4 | *Atta cephalotes h2* | -112 | -67 | 9G | GGAAGCTTCTCGAATGATCTCGAGGACTCGCGCCGTGATTGGTCA |
| 114 | hsc70-4 | *Atta cephalotes h2* | -211 | -196 | 3T | AGCCATGAATCTTCT |
| 115 | hsc70-4 | *Bombus impatiens h1* | -63 | -43 | 4G | AGAACATTCCAGAAGCGTCC |
| 116 | hsc70-4 | *Bombus impatiens h1* | -16 | -67 | 3G | AGAAGGTGCTTGAAT |
| 117 | hsc70-4 | *Bombus impatiens h2* | -14 | -2 | 3G | GGAGGTTGCCCGAAG |
| 118 | hsc70-4 | *Bombus impatiens h2* | -163 | -143 | 4T | TTTCTCGTAAAATCGTGAAA |
| 119 | hsc70-4 | *Bombus impatiens h2* | -263 | -238 | 5G | GGAACTTCCTCGTATGCTCGTGTTC |
| 120 | hsc70-4 | *Bombus terrestris h1* | -63 | -43 | 4G | AGAACATTCCAGAAGCGTCC |
| 121 | hsc70-4 | *Bombus terrestris h1* | -82 | -67 | 3G | AGAAGGTGCTTGAAT |
| 122 | hsc70-4 | *Bombus terrestris h2* | -16 | -2 | 3G | GGAGGTTGCCCGAAG |
| 123 | hsc70-4 | *Bombus terrestris h2* | -163 | -143 | 4T | TTTCTCGTAAAATCGTGAAA |
| 124 | hsc70-4 | *Bombus terrestris h2* | -263 | -238 | 5G | GGAACTTCCTCGTATGCTCGTGTTC |
| 125 | hsc70-4 | *Bombyx mori* | -55 | -35 | 4G | TGACGCATCGAGTATGTACT |
| 126 | hsc70-4 | *Bombyx mori* | -92 | -62 | 6G | GGAATGTTCCAGAATTTCCTAGTGACGTCT |
| 127 | hsc70-4 | *Camponotus floridanus h1* | -57 | -42 | 3G | AGAAGGTTCCAGAAC |
| 128 | hsc70-4 | *Camponotus floridanus h1* | -138 | -108 | 6G | CGATGACGCTCGGCCAATCGCGCGCCGGCC |
| 129 | hsc70-4 | *Camponotus floridanus h1* | -284 | -269 | 3G | GGAATTGCCGTGAAA |
| 130 | hsc70-4 | *Camponotus floridanus h1* | -326 | -306 | 4T | CGTCGCGACCGTCCGCGAAT |
| 131 | hsc70-4 | *Camponotus floridanus h1* | -408 | -383 | 5T | GCCCGCGAACCTTCTCCATCCTTCT |
| 132 | hsc70-4 | *Camponotus floridanus h1* | -564 | -549 | 3T | TTTCTGGGATCCTCT |
| 133 | hsc70-4 | *Camponotus floridanus h2* | -60 | -45 | 3G | GGAAGCTTCTCGAAG |
| 134 | hsc70-4 | *Camponotus floridanus h2* | -80 | -65 | 3T | CGCCGCGATTGGTCA |
| 135 | hsc70-4 | *Camponotus floridanus h2* | -412 | -362 | 10G | CGAGCTGTCGCGCAATTTCTCGCCATTTCTAGAAAGCACTCGAGTGATCT |
| 136 | hsc70-4 | *Camponotus floridanus h2* | -486 | -446 | 8G | TGATAAGTCGCGAGTTTTCTCGAATGTCGAAGATTTTTCT |
| 137 | hsc70-4 | *Culex quinquefasciatus h1* | -439 | -424 | 3G | TGAACATTCTAGAGA |
| 138 | hsc70-4 | *Culex quinquefasciatus h2* | -98 | -78 | 4G | GGAACCATCTGGAACGTTCT |
| 139 | hsc70-4 | *Culex quinquefasciatus h2* | -131 | -116 | 3T | TTTCCCGACGATCCG |
| 140 | hsc70-4 | *Drosophila melanogaster* | -591 | -576 | 3G | TGAAGGTCCCCGAGT |
| 141 | hsc70-4 | *Harpegnathos saltator h1* | -60 | -40 | 4G | GGAACGTTCCAGAAACTGCT |
| 142 | hsc70-4 | *Harpegnathos saltator h1* | -87 | -72 | 3G | GGTCGCAGCGGGTGC |
| 143 | hsc70-4 | *Harpegnathos saltator h1* | -140 | -120 | 4G | CGGAGATTCTCGACGTATCG |
| 144 | hsc70-4 | *Harpegnathos saltator h1* | -356 | -341 | 3T | CTACGCGACGCTTCC |
| 145 | hsc70-4 | *Harpegnathos saltator h2* | -51 | -36 | 3G | AGAAGCTTCTCGAGC |
| 146 | hsc70-4 | *Harpegnathos saltator h2* | -87 | -67 | 4G | GGCTGCTCTGAGAGACTTCT |
| 147 | hsc70-4 | *Harpegnathos saltator h2* | -200 | -175 | 5G | AGCGTGTTCTCGAATTTTCTCGTGT |
| 148 | hsc70-4 | *Harpegnathos saltator h2* | -486 | -461 | 5T | GTTCTAGAAAATTAGCGAAATCTCG |
| 149 | hsc70-4 | *Linepithema humile h1* | -61 | -41 | 4G | GGAATGTTCCAGAAGTTGCC |
| 150 | hsc70-4 | *Linepithema humile h1* | -87 | -72 | 3G | GGTCGACACGAGATC |
| 151 | hsc70-4 | *Linepithema humile h1* | -119 | -104 | 3G | CGACGATTCGTGCAG |
| 152 | hsc70-4 | *Linepithema humile h1* | -141 | -121 | 4G | CGATGCTTCTCGACCTATCG |
| 153 | hsc70-4 | *Linepithema humile h1* | -186 | -166 | 4G | AGCGTGTTCGCGGTCGTTCG |
| 154 | hsc70-4 | *Linepithema humile h1* | -412 | -397 | 3T | ACCCGCGAACATTCT |
| 155 | hsc70-4 | *Linepithema humile h2* | -57 | -37 | 4G | GGAAGCTTCTCGAGCGGGCGATTCG |
| 156 | hsc70-4 | *Linepithema humile h2* | -105 | -75 | 6G | AGAAACGACTCGTAGATTCGCGAAACCGCG |
| 157 | hsc70-4 | *Linepithema humile h2* | -130 | -115 | 3T | GTACTAGAAGGTTCG |
| 158 | hsc70-4 | *Linepithema humile h2* | -220 | -200 | 4G | AGCTCGTTCCAGTATTTTCC |
| 159 | hsc70-4 | *Linepithema humile h2* | -377 | -357 | 4T | TTTCTAGAAAATACGTGAAC |
| 160 | hsc70-4 | *Linepithema humile h2* | -522 | -507 | 3G | AGAATATTCTAGAAA |
| 161 | hsc70-4 | *Nasonia vitripennis h1* | -62 | -42 | 4G | AGAAGCTTCCAGAAGCGTCG |
| 162 | hsc70-4 | *Nasonia vitripennis h1* | -380 | -365 | 3G | CGTGTGTTCCAGAAA |
| 163 | hsc70-4 | *Nasonia vitripennis h2* | -59 | -44 | 3G | GGAAGCTTCTCGAAG |
| 164 | hsc70-4 | *Nasonia vitripennis h2* | -85 | -65 | 4G | CGGGAGCTCGCGATTGGTCG |
| 165 | hsc70-4 | *Nasonia vitripennis h2* | -178 | -163 | 3T | ATTCCAGTGTTTTCG |
| 166 | hsc70-4 | *Nasonia vitripennis h2* | -278 | -263 | 3G | AGAAACTTCTTGCAA |
| 167 | hsc70-4 | *Pogonomyrmex barbatus h1* | -56 | -36 | 4G | GGAATGTTCTAGAAGCCACG |
| 168 | hsc70-4 | *Pogonomyrmex barbatus h1* | -135 | -100 | 7G | CGAAGATTCTCGGCCAATCGCGTGCCTACCAGAAC |
| 169 | hsc70-4 | *Pogonomyrmex barbatus h1* | -233 | -218 | 3T | ACACGTGTAACTCCA |
| 170 | hsc70-4 | *Pogonomyrmex barbatus h1* | -226 | -211 | 3T | TCGCGAGAACGTTCC |
| 171 | hsc70-4 | *Pogonomyrmex barbatus h1* | -424 | -409 | 3T | TTTCTAGAACTTCTC |
| 172 | hsc70-4 | *Pogonomyrmex barbatus h2* | -58 | -43 | 3G | GGAAGCTTCTCGAAA |
| 173 | hsc70-4 | *Pogonomyrmex barbatus h2* | -91 | -76 | 3T | GCTCATGAAATCTCG |
| 174 | hsc70-4 | *Pogonomyrmex barbatus h2* | -209 | -194 | 3T | GTTCCAGTATCTTCT |
| 175 | hsc70-4 | *Pogonomyrmex barbatus h2* | -357 | -342 | 3T | CATCGCGCACTTTCC |
| 176 | hsc70-4 | *Pogonomyrmex barbatus h2* | -387 | -362 | 5G | CGCGTTTTCTGGAAAGTGCGCGAAG |
| 177 | hsc70-4 | *Pogonomyrmex barbatus h2* | -470 | -420 | 10G | GGACAAATCTCGCATTTTCTCGAATGTTCTCGAATATGCCCGAGATTTCT |
| 178 | hsc70-4 | *Pogonomyrmex barbatus h2* | -494 | -479 | 3G | TGAAACTTCTGGTCC |
| 179 | hsc70-4 | *Solenopsis invicta h1* | -57 | -37 | 4G | GGAACGTTCCAGAAGGTACA |
| 180 | hsc70-4 | *Solenopsis invicta h1* | -126 | -101 | 5G | GGACCATTCGCGCGTCCACTCGAAC |
| 181 | hsc70-4 | *Solenopsis invicta h1* | -238 | -213 | 5G | AGACCCATCTAGAAAACTCGGGAAA |
| 182 | hsc70-4 | *Solenopsis invicta h1* | -497 | -482 | 3T | GTTCATGACCGTTTC |
| 183 | hsc70-4 | *Solenopsis invicta h1* | -547 | -502 | 9T | CTTCCAGCACACACGAGAACACGCCGGAAGTTTCTAGTAGCTTCT |
| 184 | hsc70-4 | *Solenopsis invicta h2* | -59 | -44 | 3G | AGAAGCTTCTCGAAG |
| 185 | hsc70-4 | *Solenopsis invicta h2* | -109 | -79 | 6G | GGAAGCGCCTCGATGAGTCGCGAAATATCG |
| 186 | hsc70-4 | *Solenopsis invicta h2* | -231 | -216 | 3T | ATTCCAGCATCTTCC |
| 187 | hsc70-4 | *Solenopsis invicta h2* | -435 | -360 | 15T | CCTCTAGAACGATCGCGTGTTCCCTAGAAAGTGCTAGAAAGTGCTAAAAAGTACTCGAACAATCGCGAATGTTCT |
| 188 | hsc70-4 | *Tribolium castaneum* | -59 | -39 | 4G | GGAATCTTCTAGAAGCTTCA |
| 189 | hsc70-4 | *Tribolium castaneum* | -144 | -129 | 3T | TTACCGGAAGGTTCC |
| 190 | hsc70-4 | *Tribolium castaneum* | -174 | -159 | 3T | TTTCGCGAAAAATCG |
| 191 | hsc70-5 | *Acromyrmex echinatior* | -628 | -613 | 3G | CGAACGTGCTAGCAT |
| 192 | hsc70-5 | *Acromyrmex echinatior* | -485 | -470 | 3T | ATTCGTGAACACGCG |
| 193 | hsc70-5 | *Acromyrmex echinatior* | -581 | -561 | 4G | AGAACATTCGGGAAGTTTCG |
| 194 | hsc70-5 | *Acyrthosiphon pisum* | -642 | -627 | 3G | TGGATGTTCAAGATA |
| 195 | hsc70-5 | *Acyrthosiphon pisum* | -675 | -655 | 4G | AGAGCTTGCTGGAAAATCCA |
| 196 | hsc70-5 | *Atta cephalotes* | -486 | -471 | 3T | ATTCGTGAACATACG |
| 197 | hsc70-5 | *Atta cephalotes* | -582 | -562 | 4G | AGAACATTCGGGAAGTTTCG |
| 198 | hsc70-5 | *Bombus terrestris* | -24 | -9 | 3T | ATTCCGGAAACAACT |
| 199 | hsc70-5 | *Bombus terrestris* | -553 | -533 | 4G | AGGAAATTCCCGAAATGTCC |
| 200 | hsc70-5 | *Bombyx mori* | -499 | -484 | 3T | ATGCAGGAAAGCTCT |
| 201 | hsc70-5 | *Bombyx mori* | -659 | -639 | 4G | TGGCTCTTCTGTAATGTTCT |
| 202 | hsc70-5 | *Bombyx mori* | -186 | -166 | 4T | TATCTTGAAAATGCTGTAAG |
| 203 | hsc70-5 | *Camponotus floridanus* | -195 | -165 | 6G | AGAACTCTGTGGAACATTCCGGATGTTACG |
| 204 | hsc70-5 | *Culex quinquefasciatus* | -81 | -66 | 3G | CGAACTTTCGAGAGA |
| 205 | hsc70-5 | *Culex quinquefasciatus* | -399 | -379 | 4G | CGCTGCTTCTGTGATTTTCC |
| 206 | hsc70-5 | *Harpegnathos saltator* | -174 | -159 | 3T | ATTCATGAAAAAGCC |
| 207 | hsc70-5 | *Harpegnathos saltator* | -529 | -514 | 3T | AATCGCGCATTTTCG |
| 208 | hsc70-5 | *Harpegnathos saltator* | -590 | -565 | 5T | TTTCTAGAACATTCGTGATGTTTCT |
| 209 | hsc70-5 | *Pogonomyrmex barbatus* | -548 | -528 | 4G | TGAAAATTCCGTTATTATCA |
| 210 | hsc70-5 | *Solenopsis invicta* | -280 | -265 | 3G | TGAACGTTCAGGTTT |
| 211 | hsc70-5 | *Solenopsis invicta* | -683 | -663 | 4T | ATTCAAAAATTTACTTGATT |
| 212 | hsc70-5 | *Tribolium castaneum* | -509 | -494 | 3G | TGGATTTTCGGGACA |
| 213 | hsc70-5 | *Tribolium castaneum* | -367 | -352 | 3T | CTTCGAGAGTGATCG |
| 214 | hsc70-5 | *Tribolium castaneum* | -609 | -589 | 4T | CTTCGTGTGATTTCTGGATA |
| 215 | hsp60 | *Acromyrmex echinatior* | -679 | -664 | 3G | CGCACACTCGTGAAT |
| 216 | hsp60 | *Acyrthosiphon pisum* | -676 | -661 | 3T | ATTCCCGGTATTTCA |
| 217 | hsp60 | *Apis florea* | -746 | -726 | 4T | ATTCTCGTACATTCTAGAAC |
| 218 | hsp60 | *Apis mellifera* | -756 | -736 | 4T | ATTCTCGTACATTCTAGAAC |
| 219 | hsp60 | *Atta cephalotes* | -269 | -254 | 3T | ATTCGTGATAAATCG |
| 220 | hsp60 | *Atta cephalotes* | -540 | -520 | 4G | CGGAAATTCGAGAAACTTCG |
| 221 | hsp60 | *Atta cephalotes* | -603 | -583 | 4G | AGAATTTTCCCCAAGGATCG |
| 222 | hsp60 | *Bombus impatiens* | -627 | -607 | 4T | GTTCTCGTACATTCTAGAAG |
| 223 | hsp60 | *Camponotus floridanus* | -439 | -419 | 4T | TCCCGCGAAAGTTGCGGAAG |
| 224 | hsp60 | *Culex quinquefasciatus* | -464 | -444 | 4T | GTTCCAGAAGTTTCGTGGGG |
| 225 | hsp60 | *Culex quinquefasciatus* | -635 | -610 | 5T | CATCAGCCGTGTTCGTGAAGTGTGA |
| 226 | hsp60 | *Drosophila melanogaster* | -823 | -808 | 3T | TTGCCGGCAATTTCA |
| 227 | hsp60 | *Drosophila melanogaster* | -51 | -31 | 4T | TGTAATTTCCATAAGTATCC |
| 228 | hsp60 | *Linepithema humile* | -653 | -628 | 5T | CTTCTGAAAAGATCGCGAAAGTTCA |
| 229 | hsp60 | *Pogonomyrmex barbatus* | -601 | -586 | 3T | ATTCAGGATTAATCC |
| 230 | hsp60 | *Pogonomyrmex barbatus* | -767 | -752 | 3T | TTTCTAGAACCTACG |
| 231 | hsp60 | *Solenopsis invicta* | -229 | -204 | 5T | CCTCCGGAAAATTCGAGAAAGCCCC |
| 232 | hsp60 | *Tribolium castaneum* | -54 | -39 | 3T | ACTCAGGAAAGCTCC |
| 233 | hsp40 | *Acromyrmex echinatior* | -71 | -41 | 6T | ATTCTCGAAAGTTCCGGAAGCTGGTAGAAG |
| 234 | hsp40 | *Acyrthosiphon pisum* | -92 | -57 | 7T | CTTCCACGATCTTCTGGTGGATTCTAGAATATTCT |
| 235 | hsp40 | *Apis florea* | -70 | -39 | 6T | ATTCTCGAAAGTTCCGGAAGCCACTAGAAG |
| 236 | hsp40 | *Apis florea* | -346 | -325 | 4G | AGAATATTCAGCTATGTTCA |
| 237 | hsp40 | *Apis mellifera* | -69 | -39 | 6T | ATTCTCGAAAGTTCCGGAAGCCACTAGAAG |
| 238 | hsp40 | *Atta cephalotes* | -71 | -41 | 6T | ATTCTCGAAAGTTCCGGAAGCTGGTAGAAG |
| 239 | hsp40 | *Bombus impatiens* | -69 | -39 | 6T | ATTCTCGAAAGTTCCGGAAGCCACTAGAAG |
| 240 | hsp40 | *Bombus mori* | -220 | -205 | 3G | AGAAACAACGAGAAC |
| 241 | hsp40 | *Bombus terrestris* | -69 | -39 | 6T | ATTCTCGAAAGTTCCGGAAGCCACTAGAAG |
| 242 | hsp40 | *Bombyx mori* | -63 | -38 | 5G | TGAAGGTCCTAGAATGTTCTAGATG |
| 243 | hsp40 | *Camponotus floridanus* | -69 | -39 | 6T | ATTCTCGAAAGTTCCGGAAGCTGCTAGATG |
| 244 | hsp40 | *Camponotus floridanus* | -360 | -345 | 3T | ATTCACGAGAGTGCA |
| 245 | hsp40 | *Culex quinquefasciatus* | -51 | -36 | 3T | GTTCCGGGAGAGTCG |
| 246 | hsp40 | *Drosophila melanogaster* | -284 | -254 | 6T | CATCTTGAATGCTAATGAATTTTCGGGACG |
| 247 | hsp40 | *Harpegnathos saltator* | -73 | -43 | 6T | ATTCTCGAAAGTTCCGGAAGTTGCTAGATG |
| 248 | hsp40 | *Harpegnathos saltator* | -143 | -128 | 3G | TGAAATTTCGCGCCA |
| 249 | hsp40 | *Linepithema humile* | -72 | -42 | 6T | ATTCTCGAAAGTTCCGGAAGCTGCCAGATG |
| 250 | hsp40 | *Linepithema humile* | -331 | -316 | 3G | CGAATTCACGAGAAT |
| 251 | hsp40 | *Nasonia vitripennis* | -80 | -45 | 7T | CTTCGAGGCACTTCTCGAAGGTTCCGGAAAGCGCG |
| 252 | hsp40 | *Pogonomyrmex barbatus* | -97 | -67 | 6T | ATTCTCGAAAGTTCCGGAAGCTGGTAGATG |
| 253 | hsp40 | *Tribolium castaneum* | -78 | -48 | 6T | CTTCCGGAATATTCCAGAGGAGTCGCGAAT |
