# Supplementary material for: The evolution of heat shock protein sequences, cis-regulatory elements, and expression profiles in the eusocial Hymenoptera
Source: BMC Evol Biol. 2016 Jan 19;16:15. doi: 10.1186/s12862-015-0573-0 (PMC4717527; doi:10.1186/s12862-015-0573-0)
Supplement: Additional file 10: Table S3. — Primer sets for qPCR including housekeeping and heat shock genes. (DOCX 79 kb) [file 12862_2015_573_MOESM10_ESM.docx]

Table S3. Primer sets for qPCR including housekeeping and heat shock genes.

| **Gene** | **Primer 5'-3'** | **Amplicon Length (bps)** |
| --- | --- | --- |
| 18s rRNA (forward) | CTCTTTCTTGATTCGGTGGGTG |  |
| 18s rRNA (reverse) | TTAGCAGGCTAGAGTCTCGTTC | 100 |
| GAPDH (forward) | TAAGATTGCCGTCTTCAGCG |  |
| GAPDH (reverse) | ATGCCTTCTCGATGGTTGTG | 110 |
| β-actin (forward) | TAAGATTATCGCTCCACCCG |  |
| β-actin (reverse) | CTCGTCGTATTCCTGCTTCG | 112 |
| Ef1-β (forward) | GGTTCAGATGAAGAGGAAGATG |  |
| Ef1-β (reverse) | TCATCTCCCCAACTTTTCAC | 111 |
| hsp83 (forward) | AGTGCTACGAGCAATTCAGC |  |
| hsp83 (reverse) | CGGATGCAGAAGTGTGATAACG | 105 |
| hsc70-4_1 (forward) | CTTAATGTCTCCGCCGTGGATAAG |  |
| hsc70-4_1 (reverse) | CTCAGCTTCGTTTACCATCCTCTC | 115 |
| hsc70-4_2 (forward) | GATCAAGAGGAACACGACGATACC |  |
| hsc70-4_2 (reverse) | GCTCTTTCTCCCTCATAGACTTGG | 105 |
| Bip(forward) | GGTACAGTGATAGGAATTGATCTGGG |  |
| Bip(reverse) | TAAGAAGGCGTGATTCGGTTACC | 112 |
| hsc70-5 (forward) | CGTTTAGTTGGTATGCCTGC |  |
| hsc70-5 (reverse) | CAGGATCTTCAAATCTCCGTCC | 100 |
| hsp60 (forward) | GTTGAAGAAGGAATCGTTCCCG |  |
| hsp60 (reverse) | CGATCTTGATTCCAGTCTCCTG | 109 |
| hsp40 (forward) | GATATGGATCCCTTTGGACTCG |  |
| hsp40 (reverse) | CCCTTTACAAGTATTCGGACTCG | 120 |
| l2efl_#4 (forward) | TTTCCGGAGTAAGCTCGTTC |  |
| l2efl_#4 (reverse) | GACAGAAGTCTCGCATTCTTCC | 117 |
